# Astrocytic accumulation of tau fibrils isolated from Alzheimer’s disease brains induces inflammation, cell-to-cell propagation and neuronal impairment

**DOI:** 10.1186/s40478-024-01745-8

**Published:** 2024-02-26

**Authors:** Khalid Eltom, Tobias Mothes, Sylwia Libard, Martin Ingelsson, Anna Erlandsson

**Affiliations:** 1 Department of Public Health and Caring Sciences, Molecular Geriatrics, Rudbeck Laboratory, Uppsala University, Uppsala, 751 85 Sweden; 2 Department of Immunology, Genetics and Pathology, Neuro-Oncology and Neurodegeneration, Uppsala University, Uppsala, Sweden; 3 Department of Pathology, Uppsala University Hospital, Uppsala, Sweden; 4 University Health Network, Krembil Brain Institute, Toronto, ON Canada; 5 Tanz Centre for Research in Neurodegenerative Diseases, Departments of Medicine and Laboratory Medicine & Pathobiology, University of Toronto, Toronto, ON Canada

**Keywords:** Alzheimer’s disease, Tau, Astrocytes, Brain-derived fibrils, Inflammation, Neurons

## Abstract

Accumulating evidence highlights the involvement of astrocytes in Alzheimer’s disease (AD) progression. We have previously demonstrated that human iPSC-derived astrocytes ingest and modify synthetic tau fibrils in a way that enhances their seeding efficiency. However, synthetic tau fibrils differ significantly from in vivo formed fibrils. To mimic the situation in the brain, we here analyzed astrocytes’ processing of human brain-derived tau fibrils and its consequences for cellular physiology. Tau fibrils were extracted from both AD and control brains, aiming to examine any potential differences in astrocyte response depending on the origin of fibrils. Our results show that human astrocytes internalize, but fail to degrade, both AD and control tau fibrils. Instead, pathogenic, seeding capable tau proteoforms are spread to surrounding cells via tunneling nanotubes and exocytosis. Notably, accumulation of AD tau fibrils induces a stronger reactive state in astrocytes, compared to control fibrils, evident by the augmented expression of vimentin and GFAP, as well as by an increased secretion of the pro-inflammatory cytokines IL-8 and MCP-1. Moreover, conditioned media from astrocytes with AD tau fibril deposits induce synapse and metabolic impairment in human iPSC-derived neurons. Taken together, our data suggest that the accumulation of brain-derived AD tau fibrils induces a more robust inflammatory and neurotoxic phenotype in human astrocytes, accentuating the nature of tau fibrils as an important contributing factor to inflammation and neurodegeneration in AD.

**Supplementary Information:**

The online version contains supplementary material available at 10.1186/s40478-024-01745-8.

## Introduction

Alzheimer’s disease (AD) is a progressive, neurodegenerative disorder that develops over decades. The main characteristics of AD include amyloid-beta (Aβ) plaques, neurofibrillary tangles (NFT) consisting of aggregated tau, and widespread neuroinflammation [58]. However, the exact cellular and molecular mechanisms behind the propagation of AD pathology remain unclear.

Tau is a microtubule-associated protein that is predominantly expressed in neurons [4]. Under physiological conditions, tau is mostly localized to the axons where it promotes the assembly and stability of microtubules [21]. In AD and other tauopathies, hyperphosphorylation of tau induces its dissociation from microtubules [59]. Furthermore, hyperphosphorylated tau exhibits a high tendency to aggregate, forming the core of paired helical filaments (PHF) and subsequently, neuro fibrillary tangles (NFTs) [2, 26, 42]. In AD, neurofibrillar pathology appears initially in the transentorhinal region of the temporal lobe and propagates sequentially to involve the hippocampus and subsequently also the frontal, parietal, and occipital lobes [6]. Misfolded tau aggregates are known to recruit normal tau in a “prion-like” mechanism called seeding. Tau seeds have been reported in several neurodegenerative diseases, but also at lower levels in non-diseased controls [6, 10, 33, 38].

Astrocytes, the most numerous glial cell type in the brain, play a pivotal regulatory role in synaptic functioning and tissue homeostasis [56]. In addition, astrocytes are highly involved in neuroinflammation and interact extensively with all cell types in the central nervous system (CNS) [36]. Although they have none or very low endogenous expression of tau, inclusions of hyperphosphorylated tau appear frequently in astrocytes in AD and other tauopathies [43, 46]. Astrocytes have been shown to take up tau fibrils from the extracellular space, as well as through phagocytosis of tau-burdened dystrophic neurites, synapses or whole dead neurons [29, 40, 41, 53]. As a result, they undergo transformation from a homeostatic state towards a reactive state, which enhances their phagocytic behavior but hampers their neurotrophic functions [24, 45, 55]. We have previously shown that human astrocytes heavily modify ingested synthetic tau fibrils [41]. The astrocyte-modified tau fibrils have an exceptional seeding efficiency and are readily spread to neighboring astrocytes [41]. Nevertheless, synthetic tau fibrils display substantial biochemical and structural differences compared to in vivo formed fibrils [19, 54, 66]. Thus, the aim of the present study was to explore astrocytic processing and spreading of pathological tau aggregates by exposing iPSC-derived astrocytes to human brain-derived tau fibrils, as these constitute a more physiologically relevant tau proteoform.

## Materials and methods

### Culture of human iPSC-derived astrocytes

Human induced pluripotent stem cell (iPSC)-derived neuroepithelial stem (NES) cells (iPSCs, Cntrl9 II cell line) were differentiated into astrocytes following a well-established 28-day protocol with some minor changes [18, 37]. First, NES cells were cultured in cell culture flasks (Sarstedt) pre-coated with 100 µg/ml poly-L-ornithine (Sigma, P3655) and 50 µg/ml laminin (Sigma, L2020). Astrocyte differentiation medium consisted of advanced DMEM/F-12 (Thermo Fisher, 12634-010) supplemented with 1% penicillin-streptomycin (Thermo Fisher, 15140-122), 1% L-glutamine (Thermo Fisher, 25030-024), 1x non-essential amino acids (Thermo Fisher, 11,140,050), 1x B27 (Thermo Fisher, 11,530,536), 200 ng/ml IGF-1 (Sigma, SRP3069), 10 ng/ml heregulin beta-1 (Sigma, SRP3055), 10 ng/ml activin A (Peprotech, 120-14E) and 8 ng/ml bFGF (Thermo Fisher, 13,256,029). From day 15, 20 ng/ml of CNTF (Thermo Fisher, PHC7015) was also included. Culture medium was changed every other day until day 28. For experiments, fully differentiated astrocytes were detached using 4% trypsin-EDTA (Thermo Scientific, 10,779,413) and seeded at 5 000 cells/cm^2^.

### Culture of human iPSC-derived neurons

Human neurons were produced using the same NES cell line as astrocytes (iPSCs, Cntrl9 II) [8, 31]. The NES cells were seeded at a density of 40 000 cells/cm^2^ in cell culture flasks (Sarstedt) precoated with 100 µg/ml poly-L-ornithine (Sigma, P3655) and 50 µg/ml laminin (Sigma, L2020). Initially the cells were cultured in neuronal differentiation medium consisting of DMEM/F12 + Glutamax (Fisher Scientific, 31,331,028) supplemented with 1% N2 (Fisher Scientific, 11,520,536), 1% penicillin–streptomycin (Thermo Fisher, 11,548,876) and 1x B27 (Thermo Fisher, 17,504,044), with total medium change every day. At day five of differentiation, cells were detached from culture flasks using 1x TrypLE (Thermo Fisher, 12,563,029) and re-plated at a density of 20 000 cells/cm^2^ in 12-well culture plates (for immunocytochemistry) and at 60 000 cells/cm^2^ in 96-well culture plates (for the ATP assay). All plates were precoated with 100 µg/ml poly-L-ornithine (Sigma, P3655) and 250 µg/ml laminin (Sigma, L2020). For the following five days, cells were cultured in neuronal differentiation medium, with only half of the medium being replaced every other day. Thereafter, and until day 28 of differentiation, half of the medium was changed every other day with a 1:1 mixture of neuronal differentiation medium and complete neurobasal medium consisting of neurobasal medium (Thermo Fisher, 21,103,049), supplemented with 1% penicillin–streptomycin (Thermo Fisher, 11,548,876), 1x B27 (Thermo Fisher, 17,504,044) and 1x GlutaMAX (Thermo Fisher, 35,050,038).

### Extraction of human brain-derived tau fibrils

Brain tissue (400 mg) was obtained from parietal and temporal lobes of three AD patients and two controls. Tau fibrils were extracted as described by Fitzpatrick et al. with some minor modifications [19]. In short, tissue samples were homogenized in extraction buffer at 1:10 weight-to-volume ratio using Precellys® Evolution (Bertin Technologies, France). The extraction buffer consisted of 10 mM Tris-HCL, 0.8 M NaCl, 10% sucrose and 1 mM EGTA, all dissolved in Milli-Q (MQ) water, with pH correction to 7.5. Homogenates were subsequently supplemented with 2% Sarkosyl and incubated on mild shake at 37 °C for 30 min. Then, the samples were centrifuged for 10 min at 7000 g. Supernatants were collected and ultracentrifuged at 100 000 g for 60 min using a Hitachi CS150NX ultracentrifuge with a S50ST rotor. All centrifugations were performed at 4 °C. Pellets were resuspended in phosphate-buffered saline (PBS) at a concentration of 5 g starting tissue/ml. Extracted fibrils were then sonicated at 40% amplitude, 1 s on/off for a total of 1 min, and stored at -70 °C until use.

### Western blot analysis

Brain tissue extracts were denatured by incubating 5 µl of each sample with 10 µl NuPAGE™ LDS Sample Buffer (4X) (Invitrogen, NP0007), 4 µl 10X Bolt™ Sample Reducing Agent (Invitrogen, B00009) and 21 µl MQ water at 95 °C for 5 min. Samples were then loaded on 4–12% Bolt™ Bis-Tris Plus protein gels (Invitrogen, NW04125BOX), along with 5 µl PageRuler™ Plus Prestained Protein Ladder (Thermo Fisher, 26,619). The gels were run in MES SDS running buffer (Thermo Fisher, B0002) at 200 V for 15 min. Transfer from gels to PVDF membranes (Thermo Fisher, LC2005) was performed with a Power Plotter System (Thermo Fisher, PB0012) using the recommended settings for mixed range molecular weight (25 V, 1.3 A, for 7 min). The total protein concentration in each lane was quantified using the No-Stain Protein Labeling Reagent (Thermo Fisher, A44449) according to the manufacturer’s instructions. Signal intensities were measured using BIO-RAD ChemiDoc XRS. Blocking was performed by incubation in 5% Bovine Serum Albumin (BSA) in 0.1% Tris buffered saline-Tween 20 (TBS-T) at room temperature (RT) for 1 h and the membrane was then incubated overnight at 4 °C with primary antibodies. The following primary antibodies were used: Tau-5 (Invitrogen, AHB0042) to measure total tau, AT8 (Invitrogen, MN1020) to measure phosphorylated tau (pTau) at Ser202 and Thr205, Anti-tau (4-repeat isoform RD4) antibody (Sigma, 05-804) to measure 4R-tau, and Anti-Tau (3-repeat isoform RD3) antibody (Sigma, 05-803) to measure 3R-tau. All primary antibodies were diluted in 5% BSA in 0.1% TBS-T. Following washing, the membranes was incubated with secondary antibodies for 1 h at room temperature. The following secondary antibodies were used: goat anti-rabbit Dylight 680 (Invitrogen, 35,568), goat anti-mouse Dylight 800 (Invitrogen, SA5-35521) and goat anti-mouse IgG poly-HRP (Invitrogen, 32,230). All secondary antibodies were diluted in 5% BSA in 0.1% TBS-T. Enhanced chemiluminescence (ECL) signal was developed using Amersham ECL Prime Western Blotting Detection Reagent (Cytiva, RPN2232) according to manufacturer’s instructions. Flourescence and ECL signals were analyzed using SA Odyssey (LI-COR) and BIO-RAD ChemiDoc XRS, respectively. Signal intensity was measured using the ImageStudio (LI-COR) or ImageLab (BIO-RAD) softwares and normalized against the total protein blot.

### Transmission electron microscopy (TEM)

Transmission electron microscopy (TEM) with negative staining was performed to confirm the presence of tau fibrils in tissue extracts. All samples were diluted 1:3 in distilled water and then transferred onto a formvar and carbon-coated 200-mesh copper grid (Ted Pella). Samples were stained with 2% uranyl acetate and were left to dry. Dried grids were examined using TEM (FEI Tecnaii G2) operated at 80 kV with an ORIUS SC200 CCD camera and Gatan Digital Micrograph software (Gatan Inc.).

### Exposure of astrocytes to tau fibrils

Astrocytes were exposed to sonicated human brain-derived tau fibrils extracted from either AD or control brain tissue. Brain extracted fibrils were diluted to a concentration of 25 mg starting tissue/ml in culture medium. Exposure to tau fibrils lasted for 3 days, after which the cultures were washed 3 times with PBS and maintained in tau-free culture medium for an additional 12 days. The medium was replaced and astrocyte-conditioned medium (ACM) was collected at 4, 8, and 12 days post-exposure and kept at -20 °C until analysis. Astrocytes were fixed at two timepoints: 3d + 4d and 3d + 12d. Fixation was performed using 4% paraformaldehyde (Sigma) in PBS for 15 min at RT.

### Exposure of neurons to ACM

In parallel to neuronal differentiation, astrocytic cultures were either exposed to AD fibrils, control fibrils or left unexposed (as described above). Starting from day 28 of neuronal differentiation, which corresponded to 3d + 2d of astrocytic cultures, half of the neuronal culture medium was replaced every other day with a mixture of neuronal differentiation medium, complete neurobasal medium and fresh ACM at a ratio of 1:1:2, respectively. Neuronal cultures were treated for 14 days (until differentiation day 42). Then the neurons were either lysed for ATP assay or fixed using 4% paraformaldehyde (Sigma) in PBS for 15 min at RT.

### Time-lapse microscopy

Astrocytes were cultured in time-lapse culture dishes at a density of 5000 cells/cm^2^. Cells were exposed to brain-derived AD fibrils at a concentration of 25 mg starting tissue/ml in culture medium for 3 days. The cells were then washed 3 × 5 min with PBS and subsequently stained with Amytracker 680. Following a 30-minute incubation, the cells were washed 3 × 5 min with PBS and tau free culture medium was added. Cells were recorded using time-lapse microscopy (Leica DMi8 microscope). Images were captured at 40x magnification every 3 min for a total duration of 3 days.

### Immunocytochemistry

Fixed cells on cover slips were washed 3 × 5 min with PBS and blocked using 5% normal goat serum (NGS), 0.1% Triton in PBS for 1 h at RT. Then, the cells were incubated with primary antibodies (Table. S1) at RT for 4 h, washed 3 × 3 min with PBS and incubated with secondary antibodies and/or dyes at RT (Online Resource 1). All antibodies were diluted in 0.5% NGS, 0.1% Triton in PBS. After 1 h, coverslips were washed 3 × 3 min with PBS and mounted on glass slides using Ever Brite Hardset mounting medium with or without DAPI (VWR, 23,004 and 23,003).

### Measurement of cytokine concentrations in ACM

Cytokine concentrations in ACM were measured at day 3 + 4 using an electrochemiluminescence assay (Meso Scale Diagnostics). ACM from each culture condition was loaded onto a 96-well plate. A custom-designed U-Plex MSD-ECL was used to measure the concentration of following cytokines: IL-1β, IL-6, IL-8, IL-10, IL-12/IL-23p40, IL-17 A, IP-10, I-TAC, MCP-1 and TNF-α. The procedure was performed by Affinity Proteomics Uppsala and measured using a MESO SECTOR S 600MM (SciLifeLab, Uppsala University, SE-751 85 Uppsala, Sweden). Two separate astrocyte cultures were analyzed.

### Measurement of tau concentrations in ACM

Tau concentration in ACM was measured using an in-house sandwich ELISA assay. First, 96-well half-area plates were coated with Tau-5 (0.5 µg/ml; Invitrogen, MA5-12808) overnight at 4 °C. The plate was then blocked with 1% BSA in PBS for 2 h at RT. The ACM was incubated in 0.5% SDS at 95 °C for 5 min. Recombinant human 441-tau (Anaspec, AS-55,556) was used as a standard. Following overnight incubation at 4 °C, the plate was washed and biotinylated BT2 (0.5 µg/ml; Invitrogen, MN1010B) and streptavidin-HRP (1:1000; Mabtech, 3310-9-1000) were added for detection. All dilutions were made in ELISA incubation buffer (0.1% BSA and 0.05% Tween-20 in PBS). Signals were developed using K blue aqueous TMB substrate (Neogen, 331,177), stopped with 1 M H_2_SO_4_, and read with a spectrophotometer at 450 nm. Interpolation of sample concentrations was performed by plotting a second order polynomial curve using GraphPad Prism v9.0.0.

### Seeding of tau pathology

To assess the efficiency of tau fibrils to seed tau pathology, we used the tau RD P301S FRET Biosensor HEK cell line (ATCC, CRL-3275). Cells were cultured on coverslips at a density of 50 000 cells/cm^2^ in culture medium consisting of DMEM (ThermoFisher, 11,880,028), 10% fetal bovine serum (FBS), 2% Glutamax (Thermo Fisher, 35,050,038), and 1% PenStrep (Thermo Fisher, 15,140,122). The following day, FRET HEK cells were exposed to ACM from tau-exposed astrocytic cultures, supplemented with 1% lipofectamine 3000 (Fisher Scientific, L3000015) and 10% FBS (Fischer Scientific, 11,533,387). The ACM from untreated astrocyte cultures was used as a negative control. Brain extracts diluted in astrocyte culture medium at a concentration of 25 mg starting tissue/ml were used as positive controls. Both negative and positive controls were supplemented with 1% lipofectamine 3000 (Fisher Scientific, L3000015) and 10% FBS. Biosensor cells were exposed to ACM for 48 h, after which cells were fixed using 4% paraformaldehyde (Sigma) in PBS for 15 min at RT. The coverslips were washed 3 × 5 min with PBS and mounted onto glass slides using Ever Brite Hardset Mounting medium without DAPI (VWR, 23,003).

### Luminescent ATP detection assay

Human neurons were cultured in 96-well plates at 60 000 cells/cm^2^ and treated with ACM from tau-exposed astrocytic cultures as described above. Astrocytes were exposed to tau fibrils extracted from three AD and two control brains. The ACM from each culture condition was used to treat neuronal cultures for 14 days. Total ATP levels were analyzed at day 3 + 14 using the Luminescent ATP Detection Assay Kit (Abcam, ab113849) according to the manufacturer’s instructions. Luminescence was measured using Infinite M1000 plate reader (Tecan). Four separate neuronal cultures were analyzed.

### Image analysis

Fluorescent images of astrocytes were captured using the Observer Z1 Zeiss fluorescence microscope. In total, 10–12 images per condition and timepoint were captured as 30-image z-stacks using the 20x objective. Z-stacks were compiled into composites for maximum intensity and the fluorescence signal was quantified using ImageJ. For Vimentin and GFAP, the integrated density in each image was normalized to the total number of living cells. Average cell area was estimated by normalizing the total vimentin-positive area to the number of living cells in each image. Quantification of distant branching was performed using a custom-made ImageJ macro (Online Resource 2). The analysis was performed on vimentin images using the following step: convert to 8-bit, set threshold, find edge, Gaussian blur, convert to mask, skeletonize, create selection. Each selection was then put through several interactions of dilation and erosion to get the best fit possible. Branching points were calculated when pixels were in contact with 3 or more other pixels. The number of branching points within the soma was subtracted from total branching points to determine distant branching. The average number of distant branches per cell was calculated by normalizing the total number of distant branching points to the number of living cells in each image.

Quantification of the intracellular Amytracker signal was performed using a custom-made ImageJ macro (Online Resource 3). First, the region of interest (ROI) was determined using vimentin as a cellular marker to only include the intracellular Amytracker signal. Then, the following steps were applied: set scale, convert to 16-bit, subtract background, set threshold (all images were quantified using the same threshold), clear outside, set measurements (integrated density was used as a measurement of signal intensity) and analyze the signal. For each image, the total integrated density was normalized to the number of living cells.

Fluorescent images of neurons were captured using the Leica DMi8 microscope. A total of 12 images per condition were obtained as single images using the 40x objective. Quantification of the number of synaptophysin-positive puncta was performed using a custom-made ImageJ macro (Online Resource 4). First, the following steps were applied to synaptophysin images: set scale, subtract background, convert to 16-bit, set threshold, convert to mask, despeckle, watershed and analyze particles. Synaptophysin-positive puncta were counted as the number of remaining ROIs with an area of 0.4–2.6 µm^2^. The total cell area was measured using βIII-Tubulin signal. The number of synaptophysin-positive puncta was normalized to total cell area in each image.

The biosensor FRET images were captured using the 40x objective on Zeiss LSM 700 confocal microscope through the excitation of CFP (using the 405 nm laser) and detection of YFP emissions. Quantification of the FRET signal was performed as described for the Amytracker. In each image, the integrated density was normalized to total cell area (Since DAPI would interfere with CFP emissions). All image analysis was performed using ImageJ v.1.54b.

### Statistical analysis

All statistical analyses were performed using Graphpad Prism (v.9.0.0). Datasets were first tested for normal distribution, using the D’Agostino-Pearson omnibus and the Shapiro-Wilk test. Normally distributed datasets were analyzed using either unpaired t-test (for datasets with only two groups and one timepoint), one-way ANOVA (for datasets with more than two groups but only one timepoint) or two-way ANOVA (for datasets with more than two groups and two or more timepoints). Datasets that did not show normal distribution were analyzed using either the Mann–Whitney u-test (for datasets with only two groups) or Kruskal-Wallis non-parametric test (for datasets with more than two groups or timepoints). P-values were set as follows: * *p* < 0.05, ** *p* < 0.001, *** *p* < 0.0001, **** *p* < 0.00001.

## Results

### Tau fibrils were successfully extracted from human brain tissue

**Fig. 1:**
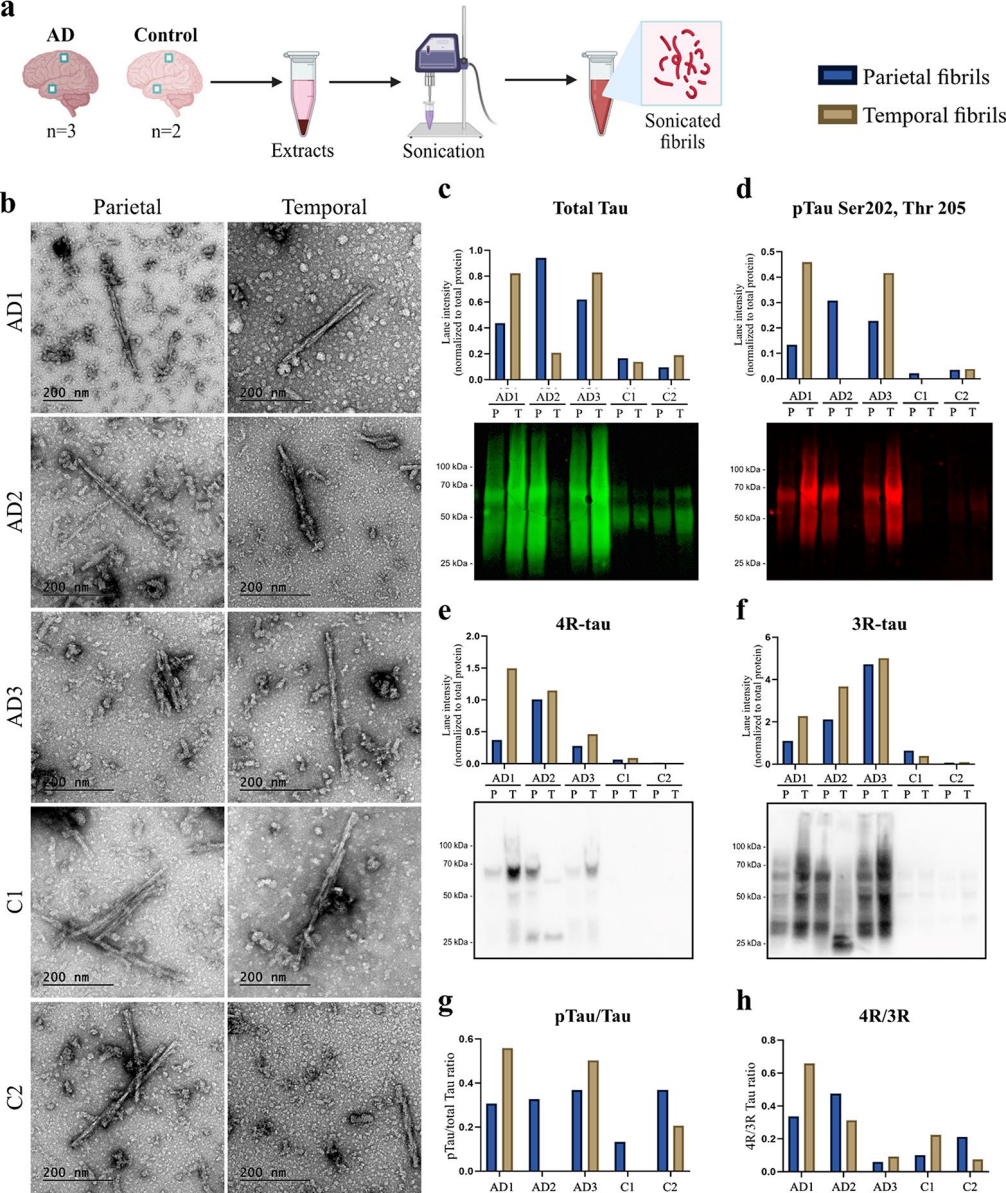
Extraction and characterization of human brain-derived tau fibrils. (**a**) Brain tissue samples were dissected from the parietal and temporal lobes of three AD patients and two controls. Extracted tau fibrils were sonicated to smaller fragments to facilitate cellular uptake. (**b**) Transmission electron microscopy (TEM) images confirmed the presence of tau fibrils in both AD and control brain tissue extracts. Western blot analysis of (**c**) total tau, (**d**) phosphorylated tau Ser202/Thr205, (**e**) 4R-tau and (**f**) 3R-tau concentrations in parietal (P) and temporal (T) samples confirmed their identity as tau fibrils. (**g**) Phospho-tau:total tau ratio and (**h**) 4R:3R tau ratio were calculated for each sample. Signal intensities in (**c**), (**d**), (**e**) and (**f**) were normalized to total protein concentration in each sample (Online Resource 6)

**Fig. 2:**
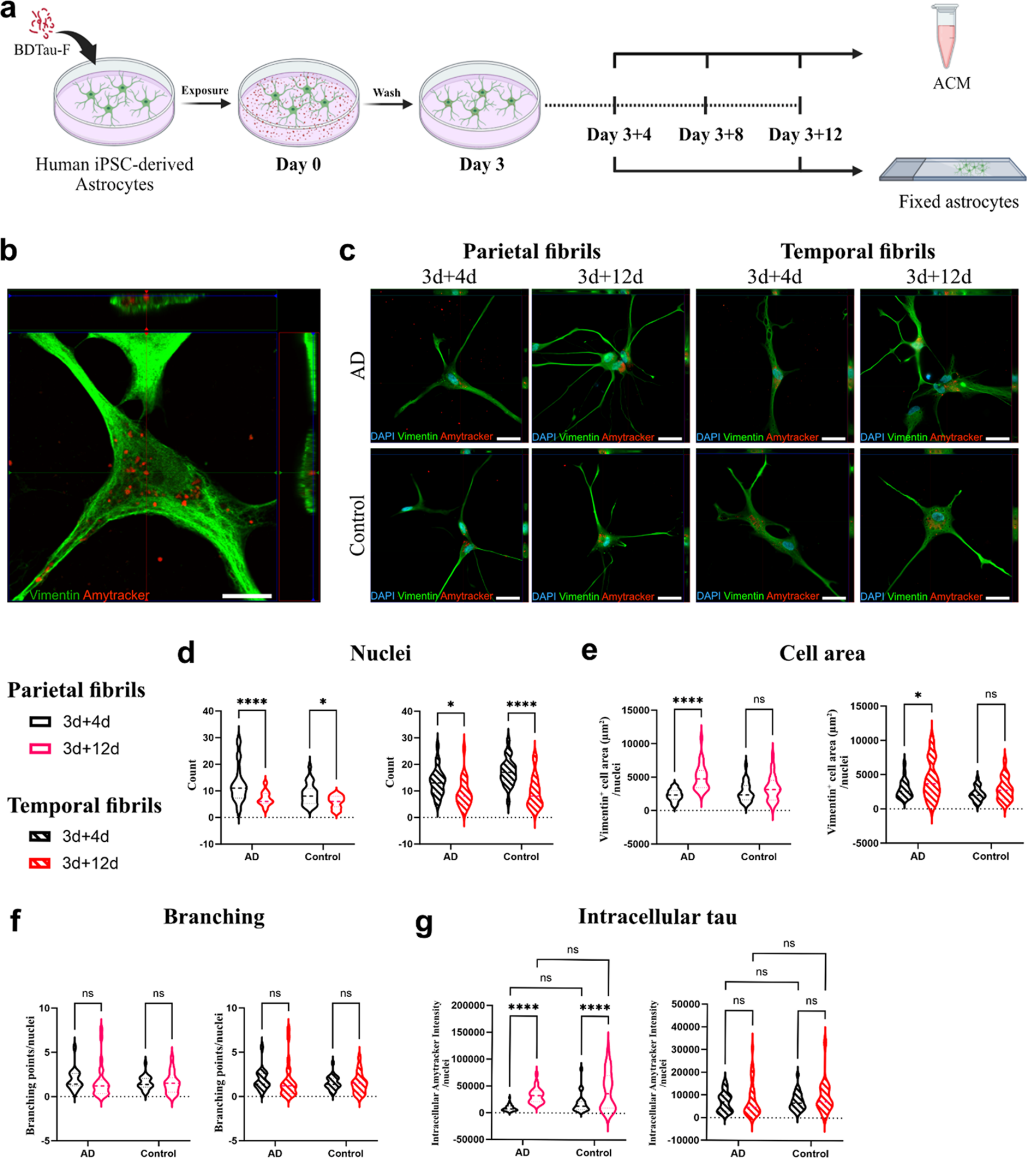
Internalized tau fibrils resist astrocytic degradation mechanisms. (**a**) Schematic illustration of the experimental design. Human iPSC-derived astrocytes were exposed to human brain-derived tau fibrils extracted from the parietal and temporal lobes of either AD or control brains. Following three days of exposure, astrocytic cultures were washed thoroughly and then maintained for an additional 12 days in tau-free medium. Astrocyte-conditioned medium was collected on day 3 + 4, 3 + 8, and 3 + 12. Astrocytes were fixed on day 3 + 4 and 3 + 12. (**b**) Confocal imaging confirmed the intracellular localization of Amytracker 680-labeled tau fibrils at day 3 + 4. Scale bar = 10 μm. (**c**) Representative fluorescence images of tau-burdened astrocytes at day 3 + 4 and 3 + 12. Scale bar = 30 μm. Quantification of cell count **(d)**, average cell area **(e)**, average number of branching points per cell **(f)** and intracellular Amytracker-680 signal **(g)** in astrocytes exposed to parietal and temporal tau fibrils. Three AD and two control samples were used. *N* = 3 culture experiments, *n* = 10–12 non-overlapping fields per brain sample. The level of significance was set at *p ≤ 0.05, **p ≤ 0.001, ***p ≤ 0.0001, ****p ≤ 0.00001

To increase the relevance of our in vitro AD model, we decided to expose human astrocytes to in vivo formed tau fibrils. For this purpose, we used *post-mortem* tissue samples from parietal and temporal lobes of three AD patients and two controls. Demographic information and clinical staging data for sampled individuals are presented in Table 1. The brain tissue was homogenized in sarkosyl, and tau fibrils were extracted and separated from contaminants through a process of differential ultracentrifugation [19] (Fig. 1a). Transmission electron microscopy (TEM) analysis confirmed the presence of fibrils in all extracts, which exhibited a twisted double-helical ribbon-like morphology with alternating width between approximately 10 and 20 nm (Online Resource 5). This aligns with the previously described morphological appearance of paired helical filaments (PHFs) [13], thus confirming successful extraction from all tissue samples (Fig. 1b). To validate the content of the fibrils, we quantified total tau, phosphorylated tau (pTau), 4R-tau and 3R-tau concentrations using western blot analysis (Fig. 1c-h). Membrane visualization revealed full-lane smears with greater intensities observed between 50 and 70 kDa (Fig. 1c-f), consistent with the previously reported description of PHFs in the brains of AD patients [23]. Quantification of signal intensities revealed higher concentrations of both total tau and pTau in AD extracts compared to controls, with an exception for the temporal AD2 sample (Fig. 2c-d). A less pronounced contrast between the two groups was observed in the pTau:total tau ratio (Fig. 2g). In addition, higher concentrations of both 4R-tau and 3R-tau were seen in AD extracts compared to controls (Fig. 2e-f). Calculation of 4R:3R ratios revealed a relative 3R-tau predominance in all AD extracts (Fig. 2h); a pattern that has been previously described in late-stage AD (BRAAK 5–6) [11, 33, 62]. Extracts from temporal lobe tissue generally contained higher concentrations of tau, pTau, 4R-tau and 3R-tau compared to parietal lobe extracts (Fig. 2c-d). This aligns with the common clinical observation that NFT pathology typically first manifests in the temporal lobes of AD patients [6]. Taken together, these observations signify that our samples are representative of typical AD disease progression.

**Table 1:** Age and clinical staging of sampled individuals

Sample	Age (years)	Dementia	BRAAK stage
AD1	84	Yes	5
AD2	86	Yes	5
AD3	74	Yes	5
Control1	88	No	0
Control2	63	No	0

### Astrocytes ingest, but fail to degrade brain derived tau fibrils

We have previously shown that astrocytes engulf in vitro produced fibrils of recombinant tau from culture medium [41]. Here, we aimed to examine the uptake and intracellular degradation of human brain-derived tau fibrils by astrocytes. As illustrated in Fig. 2a, we exposed human iPSC-derived astrocytes (Online Resource 7) to either AD or control tau fibrils for 3 days. Cultures were then thoroughly washed and maintained for up to 12 days in a tau-free medium. Astrocytes were fixed at day 3 + 4 and 3 + 12 and stained using Amytracker 680, which binds amyloid structures such as the core of tau fibrils (Online Resource 8). Consistent with our earlier findings, the astrocytes efficiently internalized substantial quantities of tau fibrils, storing them as perinuclear intracellular inclusions (Fig. 2b-c). Quantification of living nuclei revealed a significant reduction in the number of living astrocytes in both AD and control fibril-exposed cultures, suggesting a possible toxic effect of both fibril types (Fig. 2d). Morphologically, astrocytes exposed to AD fibrils demonstrated an increase in cell area over time (Fig. 2e) with a maintained number of cellular processes (Fig. 2f). However, no changes in cell area or number of processes were observed when astrocytes were exposed to control fibrils (Fig. 2e-f). Next, we aimed to investigate whether astrocytes demonstrate varying capacity to degrade AD respective control fibrils. Utilizing vimentin as a marker for cellular outlines, we compared the intracellular tau burden between day 3 + 4 and 3 + 12. Interestingly, we found no significant difference in the intracellular Amytracker signal between AD and control tau fibril-exposed astrocytes, indicating similar intracellular accumulation of both fibril types (Fig. 2g). Astrocytes exposed to parietal fibrils showed an increased intracellular signal over time, while the intracellular signal of cells exposed to temporal fibrils remained stable (Fig. 2g). The increase in intracellular tau seen for parietal fibrils is possibly explained by the observed cell death, as we have previously shown that dying astrocytes are quickly engulfed by neighboring cells, resulting in an increased load of aggregated proteins per cell [32]. Alternatively, our previous studies also show that ingested protein aggregates are brought closer together over time and eventually end up in condensed “storage dumps” around the nucleus, which leads to an increased florescence intensity [50, 57, 60, 68].

Taken together, our observations suggest that the nature of tau fibrils does not significantly affect their uptake or degradation by astrocytes. Astrocytes exhibit the capacity to readily internalize both AD and control fibrils, with limited degradation over the 12 days of intracellular storage.

### AD tau fibrils are transferred between astrocytes through tunneling nanotubes

**Fig. 3:**
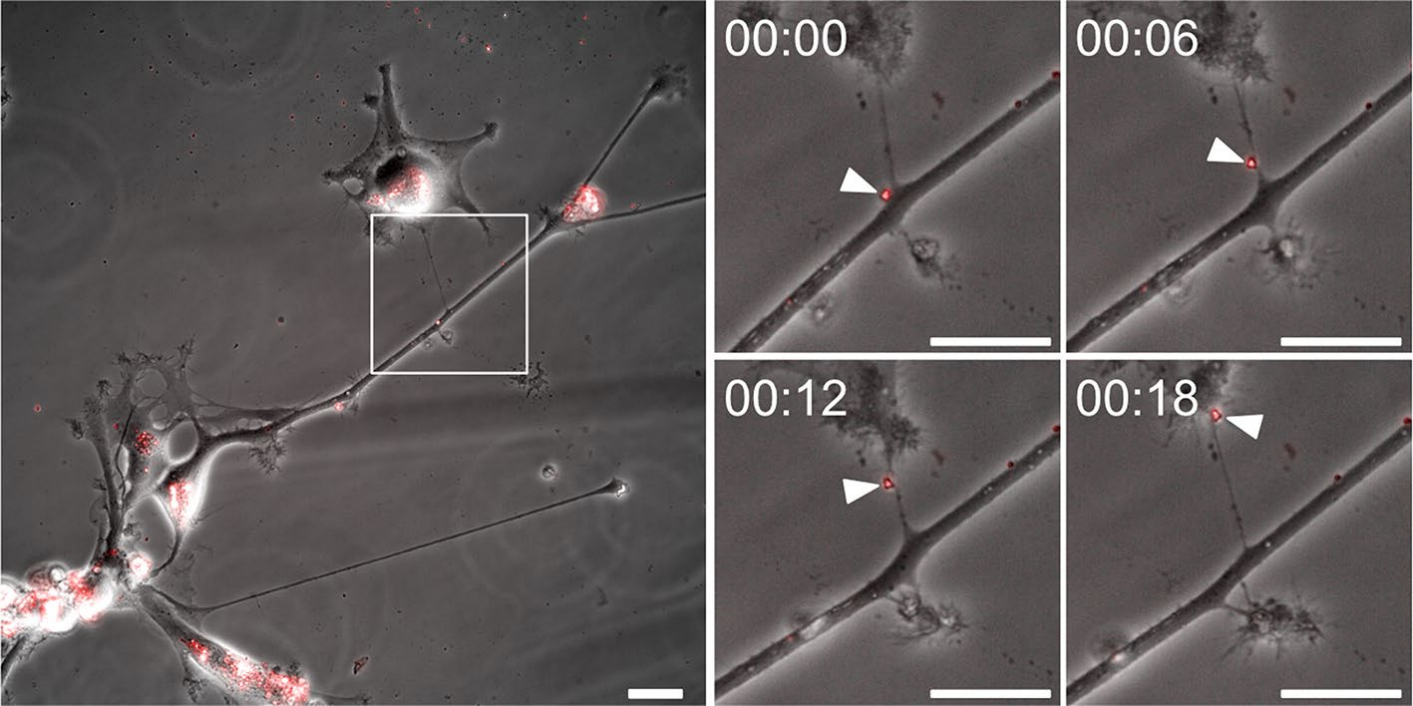
Astrocyte-to-astrocyte transfer of AD tau fibrils through tunneling nanotubes (TNTs). Time-lapse microscopy of Amytracker-labelled AD tau fibrils (shown in red) demonstrating direct cell-to-cell transmission between human astrocytes through TNTs. The presented images were captured with six-minute intervals. Arrowheads indicate a tau aggregate travelling from a projection of one astrocyte to the cell body of another. Scale bar = 25 μm

We have previously demonstrated that human astrocytes spread ingested synthetic tau and α-synuclein inclusions to neighboring astrocytes through tunneling nanotubes (TNTs) [41, 50]. Here, we aimed to validate this finding by studying direct cell-to-cell transfer of brain derived tau fibrils in cultured human astrocytes. We observed frequent TNT formation between astrocytes (Online Resource 9) at all time points, irrespective of treatment group. Using Amytracker 680 to visualize the internalized AD tau fibrils, we conducted time-lapse microscopy to detect any exchange of fibrils between astrocytes. During the three days of live imaging, we observed multiple occasions of direct cell-to-cell transfer of AD fibrils via TNTs (Fig. 3, Online Resource 10–11). Thus, astrocyte-mediated TNT transfer can potentially constitute an essential mechanism of spreading pathological tau aggregates in the human AD brain.

### Astrocytes secrete tau fibrils that seed pathology

**Fig. 4:**
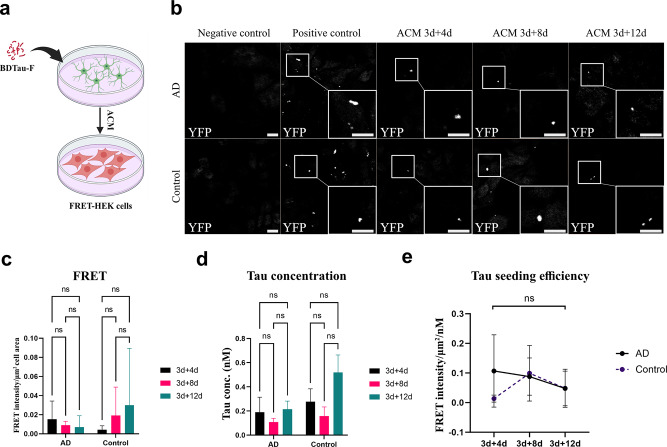
Astrocytes secrete tau fibrils with maintained seeding efficiency. (**a**) Schematic illustration of the tau seeding assay. FRET biosensor HEK cells were exposed to astrocyte-conditioned medium (ACM) from tau-exposed astrocytic cultures at day 3 + 4, 3 + 8 and 3 + 12. (**b**) Representative confocal images of ACM-exposed biosensor HEK cells. Scale bar = 10 μm. (**c**) Quantification of FRET IntDen per cell area revealed no differences across the three time points. (**d**) ELISA measurements of total tau concentrations in ACM showed no differences between the different time points. (**e**) Tau seeding efficiency presented as FRET signal normalized to tau concentration. Bars in (**c**) and (**d**), and points in (**e**), represent the means and standard deviations of measurements obtained using three AD and two control parietal samples. *N* = 3 culture experiments, *n* = 3 non-overlapping fields per brain sample. *ns*: not significant

To evaluate the seeding efficiency of tau fibrils released by astrocytes, we exposed astrocytic cultures to either AD or control tau fibrils for 3 days. All cultures were then washed thoroughly, and further cultured in tau-free medium. Astrocyte-conditioned medium (ACM) was collected at three time points: 3 + 4 days, 3 + 8 days, and 3 + 12 days and added to FRET biosensor HEK cells for 48 h. The FRET signal, detected with confocal microscopy, corresponded to the efficiency of secreted tau fibrils in ACM to induce aggregation of endogenously expressed tau monomers in HEK cells (Fig. 4a). Notably, ACM from both cultures induced tau aggregation in biosensor HEK cells (Fig. 4b, Online Resource 12). Quantitative analysis of the FRET signal unveiled no significant differences between the time points (Fig. 4c). To assess the seeding efficiency of astrocyte-secreted fibrils more precisely, we measured tau concentrations in ACM at each time point using ELISA (Fig. 4d). Normalization of the FRET signal to tau concentration confirmed that there were no differences in seeding efficiency over time, and no difference between AD fibrils and control fibrils (Fig. 4e). Based on our data, we concluded that astrocytic processing of brain-derived tau fibrils does not alter their efficiency to seed tau pathology.

### Intracellular accumulation of AD fibrils induces stronger astrocyte reactivity and pro-inflammatory state than control fibrils

**Fig. 5:**
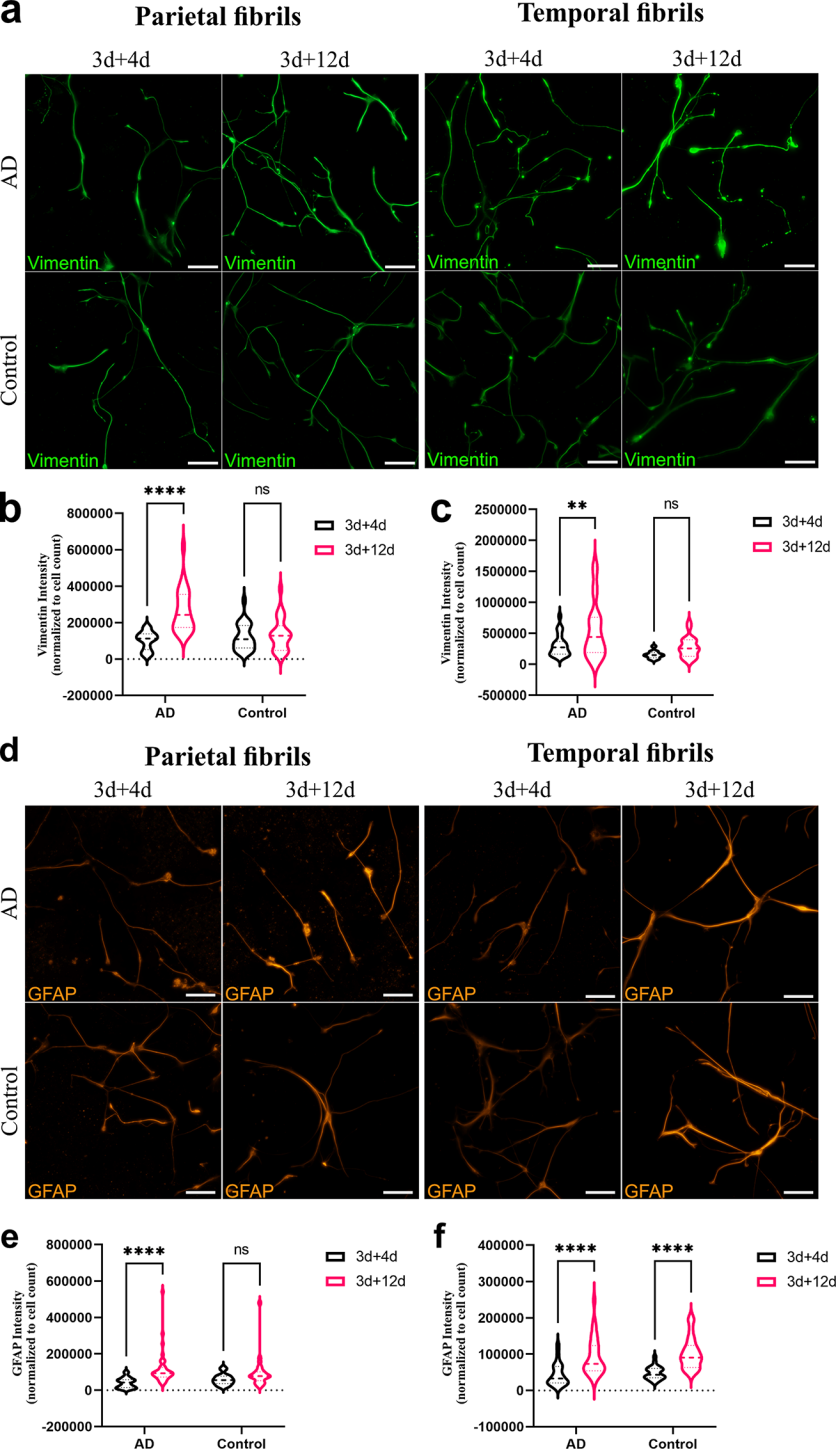
Human brain-derived tau fibrils induce a reactive state in exposed astrocytes. (**a**) Representative images of vimentin staining of astrocytes exposed to parietal and temporal tau fibrils. Quantification of vimentin IntDen in astrocytes exposed to (**b**) parietal fibrils and (**c**) temporal fibrils. (**d**) Representative images of glial fibrillary acidic protein (GFAP) staining of astrocytes. Quantification of GFAP IntDen in astrocytes exposed to (**e**) parietal fibrils and (**f**) temporal fibrils. Both for the vimentin and GFAP analysis, three AD and two control samples were used. *N* = 3 culture experiments, *n* = 10–15 non-overlapping fields per brain sample. In *b*, *c*, *e* and *f*, IntDen/image was normalized to number of nuclei. The level of significance was set at *p ≤ 0.05, **p ≤ 0.001, ***p ≤ 0.0001, ****p ≤ 0.00001. Scale bars = 100 μm

To investigate how intracellular tau deposits affect astrocytic reactivity, we studied the expression of the classical reactivity markers vimentin and glial fibrillary acidic protein (GFAP), at day 3 + 4 and day 3 + 12 post exposure. The aim was to investigate whether reactivity differed depending on which tau fibrils were added to the cultures. Vimentin was expressed by all cells at all time points (Fig. 5a). However, we observed a significant upregulation of vimentin expression in astrocytes exposed to AD fibrils from both parietal (Fig. 5b), and temporal origin (Fig. c). In contrast, there were no alterations in vimentin expression in astrocytes exposed to control fibrils (Fig. 5b-c). A similar pattern was observed for GFAP (Fig. 5d). Parietal AD fibrils led to an increased GFAP expression, while control fibrils did not (Fig. 5e). However, both AD and control temporal tau fibrils induced an up-regulation of GFAP expression (Fig. 5f).

**Fig. 6:**
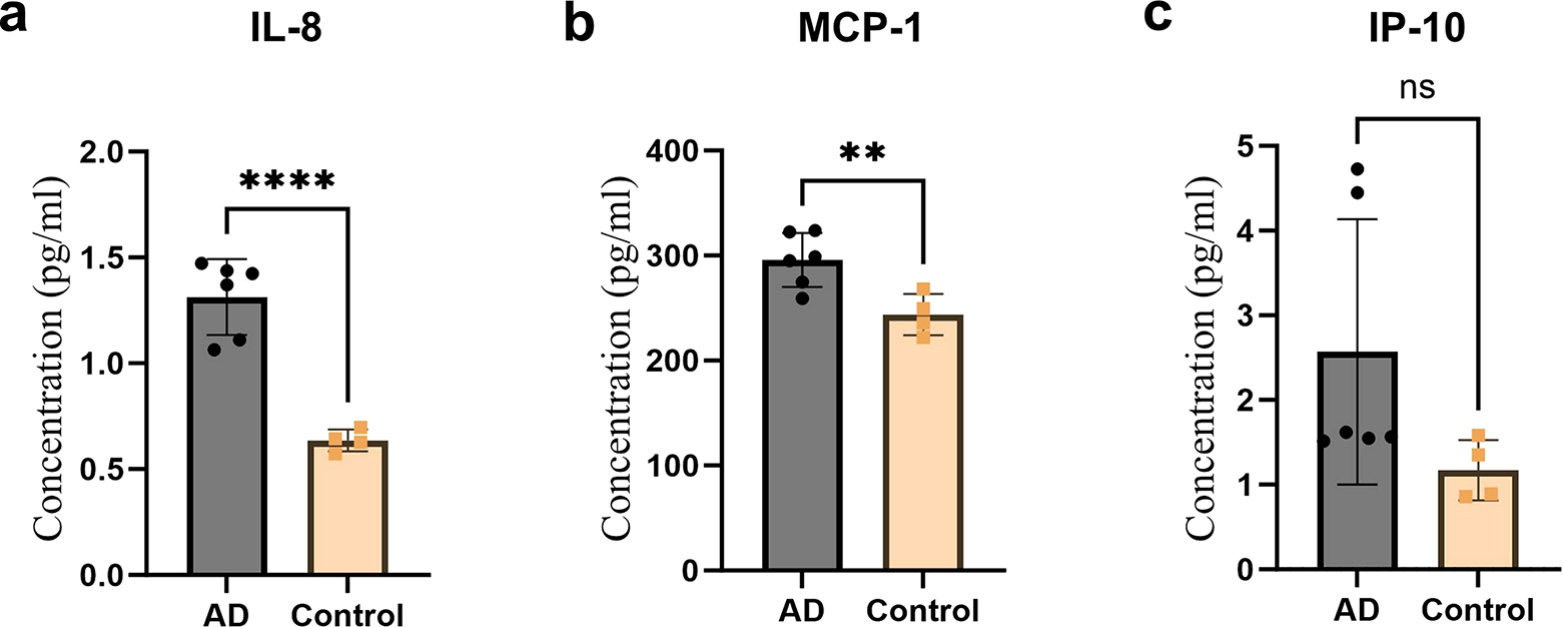
Astrocytes secrete higher levels of pro-inflammatory cytokines when exposed to AD fibrils. (**a**) IL-8, (**b**) MCP-1 and (**c**) IP-10 concentrations (pg/ml) in culture medium. All measurements were performed at day 3 + 4. Each bar represents the mean and standard deviation of measurements obtained from using three AD and two control parietal samples. *N* = 2 culture experiments, *n* = 2 measurements per brain sample. The level of significance was set at *p ≤ 0.05, **p ≤ 0.001, ***p ≤ 0.0001, ****p ≤ 0.00001

To further characterize the inflammatory state of astrocytes, we measured the concentrations of inflammatory cytokines in ACM at day 3 + 4, using a custom MSD-ECL assay. Measurements of IL-1β, IL-6, IL-10, IL-12/IL-23p40, IL-17 A, I-TAC and TNF-α resulted in below detection levels and were therefore excluded. The remaining three cytokines within range of detection were IL-8, MCP-1, and IP-10. IL-8 was detected in low concentrations of around 0.5–1.5 pg/mL (Fig. 6a), while the average MCP-1 concentration was roughly 300 folds higher (Fig. 6b). Notably, both IL-8 and MCP-1 were secreted in significantly higher concentrations when astrocytes were exposed to AD fibrils in comparison to those exposed to control fibrils (Fig. 6a-b). IP-10 concentration was comparable to that of IL-8 however, no significant differences in IP-10 levels were observed between the two groups (Fig. 6c).

Our findings collectively suggest that AD fibrils provoke a more robust reactive and inflammatory response in astrocytes in comparison to control fibrils.

### AD fibrils induce a neurotoxic phenotype in human astrocytes

**Fig. 7:**
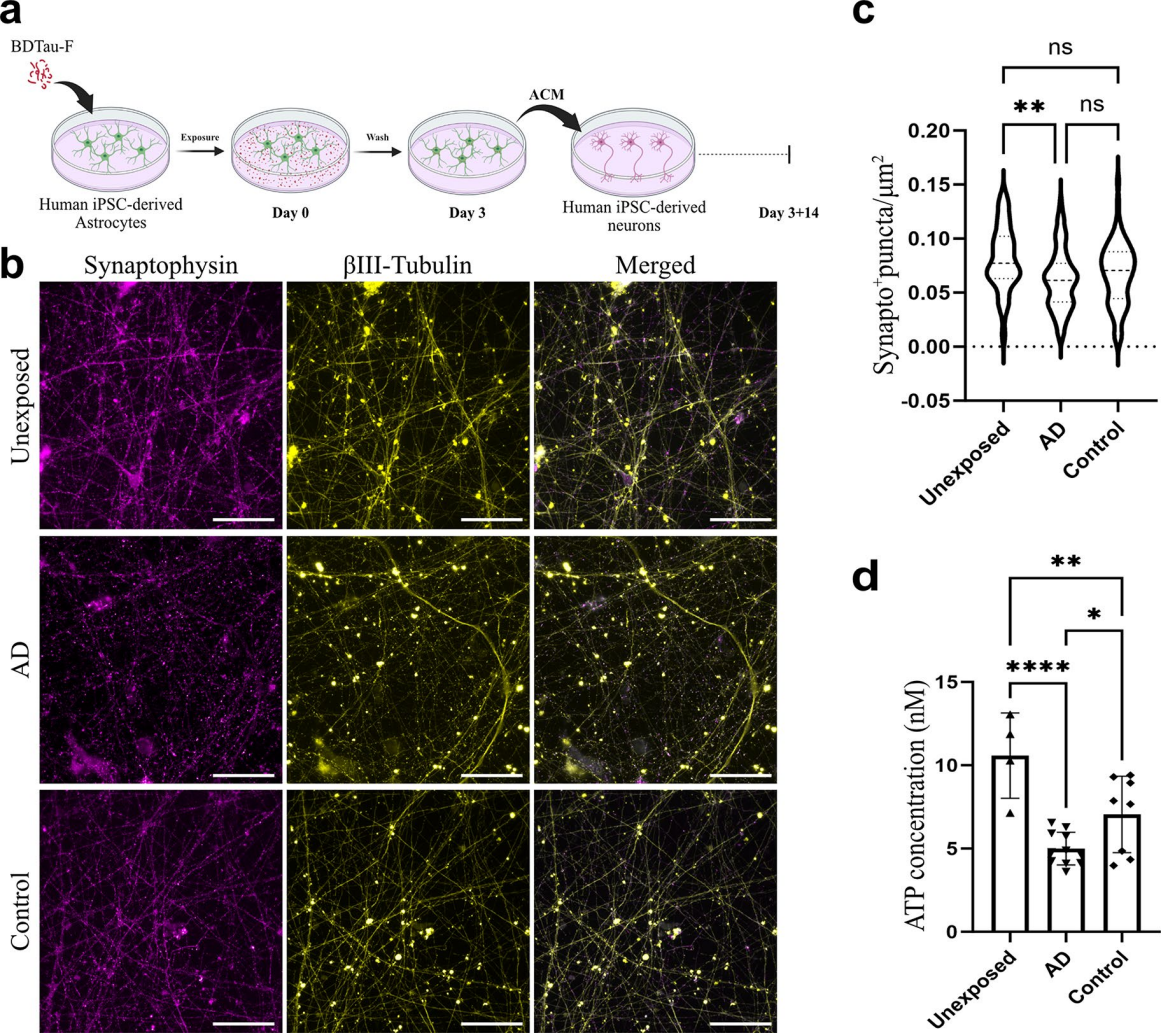
AD fibril-exposed astrocytes induce synaptic and metabolic impairment in human neurons. (**a**) Schematic illustration of the experimental design. Human iPSC-derived astrocytes were exposed to brain-derived tau fibrils. Subsequently, human iPSC-derived neurons were treated with astrocyte-conditioned medium (ACM) for 14 days. (**b**) Representative fluorescence images of synaptophysin and βIII-Tubulin staining of neurons at day 3 + 14. Scale bar = 50 μm. (**c**) Quantification of synaptophysin-positive puncta normalized to cell area (measured using βIII-Tubulin signal) at day 3 + 14 (*N* = 3 culture experiments, *n* = 10–12 non-overlapping fields per brain sample). (**d**) Measurement of ATP levels in neurons at day 3 + 14 (*N* = 4 culture experiments, *n* = 1 measurement per brain sample). Both for the synaptophysin and ATP analyses, three AD and two control parietal samples were used. Each violin plot in (**c**) represents a total of 12 images per sample. Bars in (**d**) represent the mean and SD of measurements from four separate neuronal cultures per brain sample. The level of significance was set at *p ≤ 0.05, **p ≤ 0.001, ***p ≤ 0.0001, ****p ≤ 0.0000

In AD, reactive astrocytes elicit an array of neurotoxic signatures, such as the downregulation of their neurotrophic functions and the increased secretion of pro-inflammatory factors, which promote neuroinflammation and contribute to neuronal loss [34]. Since we observed a different response to AD and control tau fibrils in terms of astrocytic reactivity and inflammation, we sought to investigate if this divergence extends to involve the astrocytes’ neurosupportive functions. To study this, we supplemented the culture medium of iPSC-derived neurons (Online Resource 10) with ACM from astrocytic cultures which had either been exposed to AD fibrils, control fibrils or were left unexposed. Our findings indicate that neurons were indeed affected by the addition of ACM (Fig. 7b). Neurons exposed to ACM from AD fibril treated astrocytes had a significantly reduced number of synaptophysin-positive puncta compared to neurons treated with ACM from unexposed astrocytes (Fig. 7c). On the other hand, no significant synaptic loss was observed in the control fibril group (Fig. 7c). Additionally, to determine if the ACM treatment affected neuronal metabolic homeostasis, we measured neuronal ATP levels. Interestingly, our data show that neurons treated with AD fibrils-ACM had significantly lower ATP levels compared to neurons treated with control fibrils-ACM (Fig. 7d), suggesting a more deleterious effect of AD tau fibrils on the capacity of astrocytes to support the metabolic demands of neurons.

Taken together, our data indicate that the accumulation of AD fibrils in human astrocytes has a more detrimental impact on their neurotrophic functions in contrast to control fibrils, as evident by their augmented synaptotoxic and metabolic effects on human neurons.

## Discussion

Astrocytes are integral to normal brain functioning. In addition, they play a central role in various neurodegenerative diseases, including AD. Astrocytic tau deposits are frequently found in tauopathies, but their significance with respect to disease propagation are poorly understood. We have previously reported that human astrocytes engulf large amounts of synthetic tau aggregates, but store rather than degrade, the ingested material [41]. In addition, astrocytes seem to act as distributors of tau seeds and spread pathology from one cell to another [41]. In the present study, we aimed to investigate astrocytic response to, and processing of, brain-derived tau fibrils from AD patients and control individuals lacking any clinical or histopathological evidence of pathology.

While in vitro-produced aggregates of recombinant tau have been the gold standard in cell culture studies, it is crucial to acknowledge that they significantly differ from the in vivo-formed tau fibrils. The interplay between alternative splicing and post-translational modifications of tau gives rise to a highly diverse pool of tau monomers in the human brain. In contrast to synthetic fibrils which are usually produced as polymers of a single, unmodified tau isoform, in vivo produced tau fibrils are composed of all six isoforms with a predominance of hyperphosphorylated tau [22, 23, 25, 28, 30, 35]. Further accentuating the divergence, electron microscopy analysis has unveiled significant structural variations among tau fibrils in different tauopathies [16–17, 67]. Nonetheless, in vitro-produced fibrils of recombinant tau fail to accurately replicate these structural conformations [66]. Thus, the strength of our current model lies in the utilization of tau fibrils extracted from human brain tissue samples, providing a more physiologically relevant representation of pathological tau aggregates.

We have previously shown that human astrocytes effectively ingest aggregates of recombinant tau, Aβ, α-synuclein, as well as whole dead cells [32, 41, 52]. However, in comparison to professional phagocytes, such as microglia and macrophages, astrocytes’ ability to degrade the ingested material is poor. To investigate the capacity of astrocytes to handle brain-derived tau fibrils, we used Amytracker 680, a fluorescent dye designed to selectively label amyloid structures such as the cores of tau fibrils. This approach holds particular significance as our previous investigations demonstrated that astrocytic modification of internalized tau fibrils influences the binding of anti-human tau antibodies [41]. Consistent with previous findings, our data show that astrocytes failed to degrade both AD and control fibrils. This observation may be elucidated by the capacity of astrocytes to function as antigen-presenting cells, as we have previously reported in our studies involving α-synuclein [51]. This role necessitates the preservation of presented proteins to ensure effective interactions with T cells and other constituents of the immune response. Moreover, several genetic diseases, collectively known as lysosomal storage disorders (LSDs) include dementia like symptoms, indicating the importance of degradation dysfunction as a contributing factor to neurodegeneration [12, 39, 61]. It has also been suggested that pathological tau aggregates can disrupt autophagy pathways, including lysosomal processing and the ubiquitin-proteosome system (UPS), thereby implying an inherent resistance to degradation [7]. Our observations emphasize the fact that human astrocytes internalize substantial quantities of fibrils from the extracellular environment and subsequently store them as intracellular perinuclear inclusions.

In the AD brain, astrocytes display certain morphological and functional alterations that signify an elevated state of reactivity. Importantly, reactive astrocytes exhibit loss of homeostatic functions and may adopt a neurotoxic phenotype, resulting in synaptic loss and secretion of pro-inflammatory cytokines [14, 63]. Hence, astrocytic reactivity is an important parameter that can influence the pathogenesis and progression of AD [9, 48]. Our analysis revealed that AD fibrils invoke a stronger reactive state in astrocytes, in comparison to control fibrils, as evident by the augmented expression of GFAP and vimentin. This was accompanied by an increased release of the inflammatory cytokines IL-8 and MCP-1. While IL-8 is predominantly a chemoattractant of neutrophils [49], MCP-1 is a potent activator and attractant of monocytes and T-cells [64]. This indicates that the interaction of astrocytes with AD tau fibrils generates a robustly pro-inflammatory milieu. Taken collectively, our findings demonstrate that tau fibrils isolated from AD brains differ from control fibrils in a way that generates differential patterns of astrocytic reactivity and inflammatory response.

Tau has been suggested to exhibit prion-like behavior, whereby misfolded tau seeds can prompt the misfolding of native tau, leading to its recruitment into intracellular inclusions that can be further transferred to neighboring cells. Existing evidence suggests that tau is transferred between neurons either directly through trans-synaptic transmission or indirectly through macropinocytosis of extracellular tau [1, 5, 20, 65]. Inter-neuronal transmission of tau is thus regarded as the primary driver for its spread across various brain regions. However, accumulating evidence indicates that phagocytic astrocytes can play a central role in tau propagation. We have previously shown that human astrocytes exchange synthetic fibrils of recombinant tau by direct transmission through TNTs [41]. In the current study, we describe a similar TNT-mediated cell-to-cell transmission of in vivo formed tau fibrils between human astrocytes, strengthening the existing evidence using a more reliable model system. Astrocytes are highly secretory cells and may re-secrete the ingested fibrils within extracellular vesicles or as pure aggregates. We have previously demonstrated that astrocytic processing of synthetic tau fibrils enhances their seeding efficiency [41]. To elucidate whether this phenomenon extends to brain-derived fibrils, we exposed RD tau P301S FRET biosensor HEK cells to astrocyte-conditioned culture medium. Indeed, astrocytes with deposits of brain-derived tau fibrils re-secrete seeding-prone tau species to the media. However, contrary to our previous findings, astrocytic processing of human brain-derived tau fibrils does not appear to impact their seeding efficiency. We hypothesize that this can be attributed to prior processing of the fibrils within the brain (possibly by astrocytes), achieving a maximized level of seeding efficiency that is resistant to further enhancement. In summary, our findings support the notion that astrocytes actively participate in the dissemination of pathological tau aggregates across distinct brain regions.

Synaptic loss is a central feature of AD and is likely to contribute to the cognitive decline that manifests in patients. Since astrocytes play a fundamental role in synapse formation, maintenance, and elimination in the healthy brain, they are most likely implicated in the synaptic damage observed during AD. The tau-burdened astrocytes could affect the neuronal functionality directly by altered phagocytosis and pruning of synapses or indirectly by failing to maintain neurosupportive functions [27]. Here, we demonstrate that ACM indeed induces a marked synaptotoxic effect on human neurons when astrocytes are exposed to AD fibrils, but not to control fibrils. Similarly, neuronal ATP levels are severely reduced when neurons are exposed to ACM from AD fibril-burdened astrocytes, implying a critical metabolic dysfunction in neurons. We hypothesize that the different patterns of neuronal impairment are potentially a product of the varying degrees of astrocytic reactivity between the two culture conditions, since several studies have consistently demonstrated a negative correlation between astrocytic reactivity and the number of neuronal synapses in AD [3, 44, 47]. Moreover, the observed synaptic and metabolic effects are presumably a consequence of altered astrocytic secretome in response to tau fibrils, since astrocytes were not in direct contact with neurons in our experimental setup.

In conclusion, we show that astrocytes possess remarkable efficiency in the internalization and subsequent re-secretion of highly pathogenic tau fibrils, thereby potentially seeding pathology in new cells in a prion-like manner. Our findings also suggest that tau fibrils isolated from AD patients differ from control fibrils in a manner that elicits distinct patterns of astrocytic reactivity, inflammatory response and neuronal impairment. This highlights the importance of using disease relevant tau species when studying AD and other tauopathies, since disease-specific variations in primary protein folding or fibrillar structure may significantly influence the developing pathology.

### Electronic supplementary material

Below is the link to the electronic supplementary material.

**Online Resource 1**. List of all antibodies and dyes used in this study.

**Online Resource 2**. Macro for quantification of astrocytes’ distant branching points.

**Online Resource 3**. Macro for quantification of intracellular Amytracker signal.

**Online Resource 4**. Macro for quantification of Synaptophysin-positive puncta.

**Online Resource 5**. Transmission electron microscopy (TEM) measurements of tau fibrils.

**Online Resource 6**. Full western blots and total protein (No-Stain) blots.

**Online Resource 7**. Human iPSC derived astrocytes express cell type-specific markers.

**Online Resource 8**. Fluorescent images and quantification of intracellular Amytracker signal in astrocytes.

**Online Resource 9**. Confocal imaging of phalloidin-stained human astrocytes demonstrating tunneling nanotube (TNT) formation.

**Online Resource 10**. Movie demonstrating tunneling nanotube (TNT)-mediated transfer of Amytracker-labelled tau fibril between human astrocytes.

**Online Resource 10**. Movie demonstrating tunneling nanotube (TNT)-mediated transfer of Amytracker-labelled tau fibril between human astrocytes.

**Online Resource 12**. Confocal images of astrocyte-conditioned medium (ACM)-exposed biosensor HEK cells.

**Online Resource 13**. Human iPSC derived neurons express cell type-specific markers.

## Data Availability

Not applicable.

## References

[1] Ahmed Z, Cooper J, Murray TK, Garn K, McNaughton E, Clarke H, Parhizkar S, Ward MA, Cavallini A, Jackson S, Bose S, Clavaguera F, Tolnay M, Lavenir I, Goedert M, Hutton ML, O’Neill MJ (2014). A novel in vivo model of tau propagation with rapid and progressive neurofibrillary tangle pathology: the pattern of spread is determined by connectivity, not proximity. Acta Neuropathol.

[2] Alonso ADC, Zaidi T, Novak M, Grundke-Iqbal I, Iqbal K (2001). Hyperphosphorylation induces self-assembly of tau into tangles of paired helical filaments/straight filaments. Proc Natl Acad Sci U S A.

[3] Bachstetter AD, Norris CM, Sompol P, Wilcock DM, Goulding D, Neltner JH, Daret S, Watterson DM, van Eldik LJ (2012). Early Stage Drug Treatment that normalizes Proinflammatory Cytokine Production attenuates synaptic dysfunction in a mouse model that exhibits age-dependent progression of Alzheimer’s Disease-Related Pathology. J Neurosci.

[4] Binder LI, Frankfurter A, Rebhun LI (1985). The distribution of tau in the mammalian central nervous system. J Cell Biol.

[5] Braak H, Del Tredici K (2011). Alzheimer’s pathogenesis: is there neuron-to-neuron propagation?. Acta Neuropathol.

[6] Braak H, Thal DR, Ghebremedhin E, Del Tredici K (2011). Stages of the pathologic process in Alzheimer Disease: age categories from 1 to 100 years. J Neuropathol Exp Neurol.

[7] Caballero B, Wang Y, Diaz A, Tasset I, Juste YR, Stiller B, Mandelkow EM, Mandelkow E, Cuervo AM (2018). Interplay of pathogenic forms of human tau with different autophagic pathways. Aging Cell.

[8] Calvo-Garrido J, Winn D, Maffezzini C, Wedell A, Freyer C, Falk A, Wredenberg A (2021). Protocol for the derivation, culturing, and differentiation of human iPS-cell-derived neuroepithelial stem cells to study neural differentiation in vitro. STAR Protoc.

[9] Ceyzériat K, Ben Haim L, Denizot A, Pommier D, Matos M, Guillemaud O, Palomares MA, Abjean L, Petit F, Gipchtein P, Gaillard MC, Guillermier M, Bernier S, Gaudin M, Aurégan G, Joséphine C, Déchamps N, Veran J, Langlais V, Cambon K, Bemelmans AP, Baijer J, Bonvento G, Dhenain M, Deleuze JF, Oliet SHR, Brouillet E, Hantraye P, Carrillo-de Sauvage MA, Olaso R, Panatier A, Escartin C (2018). Modulation of astrocyte reactivity improves functional deficits in mouse models of Alzheimer’s disease. Acta Neuropathol Commun.

[10] Chatterjee S, Sealey M, Ruiz E, Pegasiou CM, Brookes K, Green S, Crisford A, Duque-Vasquez M, Luckett E, Robertson R, Richardson P, Vajramani G, Grundy P, Bulters D, Proud C, Vargas-Caballero M, Mudher A (2023). Age-related changes in tau and autophagy in human brain in the absence of neurodegeneration. PLoS ONE.

[11] Cherry JD, Esnault CD, Baucom ZH, Tripodis Y, Huber BR, Alvarez VE, Stein TD, Dickson DW, McKee AC (2021). Tau isoforms are differentially expressed across the hippocampus in chronic traumatic encephalopathy and Alzheimer’s disease. Acta Neuropathol Commun.

[12] Clarke J, Kayatekin C, Viel C, Shihabuddin L, Sardi SP (2021). Murine models of lysosomal storage diseases exhibit differences in brain protein aggregation and neuroinflammation. Biomedicines.

[13] Crowther RA (1991). Straight and paired helical filaments in Alzheimer disease have a common structural unit (neuroflIriary tangles/neuropathdology/antibody labeling/electron microscopy/image processing). Proc Nati Acad Sci USA.

[14] Dai DL, Li M, Lee EB (2023). Human Alzheimer’s disease reactive astrocytes exhibit a loss of homeostastic gene expression. Acta Neuropathol Commun.

[15] Falcon B, Zhang W, Schweighauser M, Murzin AG, Vidal R, Garringer HJ, Ghetti B, Scheres SHW, Goedert M (2018). Tau filaments from multiple cases of sporadic and inherited Alzheimer’s disease adopt a common Fold. Acta Neuropathol.

[16] Falcon B, Zhang W, Murzin AG, Murshudov G, Garringer HJ, Vidal R, Crowther RA, Ghetti B, Scheres SHW, Goedert M (2018). Structures of filaments from pick’s disease reveal a novel tau protein fold. Nat 2018.

[17] Falcon B, Zivanov J, Zhang W, Murzin AG, Garringer HJ, Vidal R, Crowther RA, Newell KL, Ghetti B, Goedert M, Scheres SHW (2019). Novel tau filament fold in chronic traumatic encephalopathy encloses hydrophobic molecules. Nature.

[18] Falk A, Koch P, Kesavan J, Takashima Y, Ladewig J, Alexander M, Wiskow O, Tailor J, Trotter M, Pollard S, Smith A, Brüstle O (2012). Capture of Neuroepithelial-Like Stem cells from pluripotent stem cells provides a versatile system for in Vitro production of human neurons. PLoS ONE.

[19] Fitzpatrick AWP, Falcon B, He S, Murzin AG, Murshudov G, Garringer HJ, Crowther RA, Ghetti B, Goedert M, Scheres SHW (2017). Cryo-EM structures of tau filaments from Alzheimer’s disease. Nat 2017.

[20] Frost B, Jacks RL, Diamond MI (2009). Propagation of tau misfolding from the outside to the inside of a cell. J Biol Chem.

[21] Goedert M, Jakes R (1990). Expression of separate isoforms of human tau protein: correlation with the tau pattern in brain and effects on tubulin polymerization. EMBO J.

[22] Goedert M, Wischik CM, Crowther RA, Walker JE, Klug A (1988). Cloning and sequencing of the cDNA encoding a core protein of the paired helical filament of Alzheimer disease: identification as the microtubule-associated protein tau. Proc Natl Acad Sci.

[23] Goedert M, Spillantini MG, Cairns NJ, Crowther RA (1992). Tau proteins of Alzheimer Paired Helical filaments: abnormal phosphorylation of all six brain lsoforms I. Neuron.

[24] Gomez-Arboledas A, Davila JC, Sanchez-Mejias E, Navarro V, Nuñez-Diaz C, Sanchez-Varo R, Sanchez-Mico MV, Trujillo-Estrada L, Fernandez-Valenzuela JJ, Vizuete M, Comella JX, Galea E, Vitorica J, Gutierrez A (2018). Phagocytic clearance of presynaptic dystrophies by reactive astrocytes in Alzheimer’s disease. Glia.

[25] Greenberg SG, Davies P, Schein JD, Binder LI (1992). Hydrofluoric acid-treated tau PHF proteins display the same biochemical properties as normal tau. J Biol Chem.

[26] Grundke-Iqbal I, Iqbal K, Tung YC, Quinlan M, Wisniewski HM, Binder LI (1986) Abnormal phosphorylation of the microtubule-associated protein tau (tau) in Alzheimer cytoskeletal pathology. Proceedings of the National Academy of Sciences 83:4913–4917. 10.1073/PNAS.83.13.4913 10.1073/pnas.83.13.4913 PMC323854 3088567

[27] Hulshof LA, van Nuijs D, Hol EM, Middeldorp J (2022). The role of astrocytes in synapse loss in Alzheimer’s Disease: a systematic review. Front Cell Neurosci.

[28] Ihara Y, Nukina N, Miura R, Ogawara M (1986). Phosphorylated tau protein is integrated into paired helical filaments in Alzheimer’s disease. J Biochem.

[29] Ikeda K, Haga C, Akiyama H, Kase K, Iritani S (1992). Coexistence of paired helical filaments and glial filaments in astrocytic processes within ghost tangles. Neurosci Lett.

[30] Kondo J, Honda T, Mori H, Hamada Y, Miura R, Ogawara M, Ihara Y (1988). The carboxyl third of tau is tightly bound to paired helical filaments. Neuron.

[31] Konstantinidis E, Portal B, Mothes T, Beretta C, Lindskog M, Erlandsson A (2023). Intracellular deposits of amyloid-beta influence the ability of human iPSC-derived astrocytes to support neuronal function. J Neuroinflammation.

[32] Konstantinidis E, Dakhel A, Beretta C, Erlandsson A (2023). Long-term effects of amyloid-beta deposits in human iPSC-derived astrocytes. Mol Cell Neurosci.

[33] Lace G, Savva GM, Forster G, De Silva R, Brayne C, Matthews FE, Barclay JJ, Dakin L, Ince PG, Wharton SB (2009). Hippocampal tau pathology is related to neuroanatomical connections: an ageing population-based study. Brain.

[34] Lawrence JM, Schardien K, Wigdahl B, Nonnemacher MR (2023) Roles of neuropathology-associated reactive astrocytes: a systematic review. Acta Neuropathologica Communications 2023 11:1 11:1–28. 10.1186/S40478-023-01526-9 10.1186/s40478-023-01526-9 PMC10009953 36915214

[35] Lee VMY, Balin BJ, Otvos L, Trojanowski JQ (1991) A68: a Major Subunit of Paired Helical Filaments and Derivatized Forms of Normal Tau. Science (1979) 251:675–678. 10.1126/SCIENCE.1899488 10.1126/science.1899488 1899488

[36] Linnerbauer M, Wheeler MA, Quintana FJ (2020). Astrocyte crosstalk in CNS inflammation. Neuron.

[37] Lundin A, Delsing L, Clausen M, Ricchiuto P, Sanchez J, Sabirsh A, Ding M, Synnergren J, Zetterberg H, Brolén G, Hicks R, Herland A, Falk A (2018). Human iPS-Derived astroglia from a stable neural precursor State Show Improved Functionality compared with conventional astrocytic models. Stem Cell Rep.

[38] Manca M, Standke HG, Browne DF, Huntley ML, Thomas OR, Orrú CD, Hughson AG, Kim Y, Zhang J, Tatsuoka C, Zhu X, Hiniker A, Coughlin DG, Galasko D, Kraus A (2023). Tau seeds occur before earliest Alzheimer’s changes and are prevalent across neurodegenerative diseases. Acta Neuropathol.

[39] Marques ARA, Saftig P (2019) Lysosomal storage disorders– challenges, concepts and avenues for therapy: beyond rare diseases. J Cell Sci 132. 10.1242/JCS.221739/57262 10.1242/jcs.221739 30651381

[40] Martini-Stoica H, Cole AL, Swartzlander DB, Chen F, Wan YW, Bajaj L, Bader DA, Lee VMY, Trojanowski JQ, Liu Z, Sardiello M, Zheng H (2018). TFEB enhances astroglial uptake of extracellular tau species and reduces tau spreading. J Exp Med.

[41] Mothes T, Portal B, Konstantinidis E, Eltom K, Libard S, Streubel-Gallasch L, Ingelsson M, Rostami J, Lindskog M, Erlandsson A (2023). Astrocytic uptake of neuronal corpses promotes cell-to-cell spreading of tau pathology. Acta Neuropathol Commun.

[42] Noble W, Planel E, Zehr C, Olm V, Meyerson J, Suleman F, Gaynor K, Wang L, LaFrancois J, Feinstein B, Burns M, Krishnamurthy P, Wen Y, Bhat R, Lewis J, Dickson D, Duff K (2005). Inhibition of glycogen synthase kinase-3 by lithium correlates with reduced tauopathy and degeneration in vivo. Proc Natl Acad Sci U S A.

[43] Nolan A, De Paula Franca Resende E, Petersen C, Neylan K, Spina S, Huang E, Seeley W, Miller Z, Grinberg LT (2019). Astrocytic tau deposition is frequent in typical and atypical Alzheimer Disease presentations. J Neuropathol Exp Neurol.

[44] Paradisi S, Sacchetti B, Balduzzi M, Gaudi S, Malchiodi-Albedi F (2004). Astrocyte modulation of in vitro β-amyloid neurotoxicity. Glia.

[45] Piacentini R, Li Puma DD, Mainardi M, Lazzarino G, Tavazzi B, Arancio O, Grassi C (2017). Reduced gliotransmitter release from astrocytes mediates tau-induced synaptic dysfunction in cultured hippocampal neurons. Glia.

[46] Probst A, Ulrich J, Heitz PU (1982). Senile dementia of Alzheimer type: astroglial reaction to extracellular neurofibrillary tangles in the hippocampus. An immunocytochemical and electron-microscopic study. Acta Neuropathol.

[47] Qiao J, Wang J, Wang H, Zhang Y, Zhu S, Adilijiang A, Guo H, Zhang R, Guo W, Luo G, Qiu Y, Xu H, Kong J, Huang Q, Li XM (2016). Regulation of astrocyte pathology by fluoxetine prevents the deterioration of Alzheimer phenotypes in an APP/PS1 mouse model. Glia.

[48] Reichenbach N, Delekate A, Plescher M, Schmitt F, Krauss S, Blank N, Halle A, Petzold GC (2019). Inhibition of Stat3-mediated astrogliosis ameliorates pathology in an Alzheimer’s disease model. EMBO Mol Med.

[49] Roebuck KA (2004) Regulation of Interleukin-8 gene expression. 19(429–438). 10.1089/107999099313866. https://home.liebertpub.com/jir 10.1089/107999099313866 10386854

[50] Rostami J, Holmqvist S, Lindström V, Sigvardson J, Westermark GT, Ingelsson M, Bergström J, Roybon L, Erlandsson A (2017). Human astrocytes transfer aggregated alpha-synuclein via tunneling nanotubes. J Neurosci.

[51] Rostami J, Fotaki G, Sirois J, Mzezewa R, Bergström J, Essand M, Healy L, Erlandsson A (2020) Astrocytes have the capacity to act as antigen-presenting cells in the Parkinson’s disease brain. J Neuroinflammation 17. 10.1186/S12974-020-01776-7 10.1186/s12974-020-01776-7 PMC7164247 32299492

[52] Rostami J, Mothes T, Kolahdouzan M, Eriksson O, Moslem M, Bergström J, Ingelsson M, O’Callaghan P, Healy LM, Falk A, Erlandsson A (2021). Crosstalk between astrocytes and microglia results in increased degradation of α-synuclein and amyloid-β aggregates. J Neuroinflammation.

[53] Sanchez-Mico MV, Jimenez S, Gomez-Arboledas A, Muñoz-Castro C, Romero-Molina C, Navarro V, Sanchez-Mejias E, Nuñez-Diaz C, Sanchez-Varo R, Galea E, Davila JC, Vizuete M, Gutierrez A, Vitorica J (2021). Amyloid-β impairs the phagocytosis of dystrophic synapses by astrocytes in Alzheimer’s disease. Glia.

[54] Shi Y, Zhang W, Yang Y, Murzin AG, Falcon B, Kotecha A, van Beers M, Tarutani A, Kametani F, Garringer HJ, Vidal R, Hallinan GI, Lashley T, Saito Y, Murayama S, Yoshida M, Tanaka H, Kakita A, Ikeuchi T, Robinson AC, Mann DMA, Kovacs GG, Revesz T, Ghetti B, Hasegawa M, Goedert M, Scheres SHW (2021). Structure-based classification of tauopathies. Nature.

[55] Sidoryk-Wegrzynowicz M, Gerber YN, Ries M, Sastre M, Tolkovsky AM, Spillantini MG (2017). Astrocytes in mouse models of tauopathies acquire early deficits and lose neurosupportive functions. Acta Neuropathol Commun.

[56] Sofroniew MV, Vinters HV (2010). Astrocytes: Biology and pathology. Acta Neuropathol.

[57] Söllvander S, Nikitidou E, Brolin R, Söderberg L, Sehlin D, Lannfelt L, Erlandsson A (2016). Accumulation of amyloid-β by astrocytes result in enlarged endosomes and microvesicle-induced apoptosis of neurons. Mol Neurodegener.

[58] Stelzmann RA, Norman Schnitzlein H, Reed Murtagh F (1995). An English translation of alzheimer’s 1907 paper, über eine Eigenartige Erkankung Der Hirnrinde. Clin Anat.

[59] Stokin GB, Lillo C, Falzone TL, Brusch RG, Rockenstein E, Mount SL, Raman R, Davies P, Masliah E, Williams DS, Goldstein LSB (2005). Axonopathy and transport deficits early in the pathogenesis of Alzheimer’s disease. Science.

[60] Streubel-Gallasch L, Giusti V, Sandre M, Tessari I, Plotegher N, Giusto E, Masato A, Iovino L, Battisti I, Arrigoni G, Shimshek D, Greggio E, Tremblay ME, Bubacco L, Erlandsson A, Civiero L (2021). Parkinson’s Disease-Associated LRRK2 interferes with Astrocyte-Mediated Alpha-Synuclein Clearance. Mol Neurobiol.

[61] Suzuki K, Parker CC, Pentchev PG, Katz D, Ghetti B, D’Agostino AN, Carstea ED (1995). Neurofibrillary tangles in Niemann-pick disease type C. Acta Neuropathol.

[62] Uematsu M, Nakamura A, Ebashi M, Hirokawa K, Takahashi R, Uchihara T (2018). Brainstem tau pathology in Alzheimer’s disease is characterized by increase of three repeat tau and independent of amyloid β. Acta Neuropathol Commun.

[63] Wasilewski D (2022). Reactive astrocytes contribute to Alzheimer’s Disease-related neurotoxicity and synaptotoxicity in a neuron-astrocyte co-culture assay. Front Cell Neurosci.

[64] Weiss JM, Berman JW (1998). Astrocyte expression of monocyte chemoattractant protein-1 is differentially regulated by transforming growth factor beta. J Neuroimmunol.

[65] Wu JW, Herman M, Liu L, Simoes S, Acker CM, Figueroa H, Steinberg JI, Margittai M, Kayed R, Zurzolo C, Di Paolo G, Duff KE (2013). Small misfolded tau species are internalized via bulk endocytosis and anterogradely and retrogradely transported in neurons. J Biol Chem.

[66] Zhang W, Falcon B, Murzin AG, Fan J, Crowther RA, Goedert M, Scheres SHW (2019). Heparin-induced tau filaments are polymorphic and differ from those in Alzheimer’s and pick’s diseases. Elife 8.

[67] Zhang W, Tarutani A, Newell KL, Murzin AG, Matsubara T, Falcon B, Vidal R, Garringer HJ, Shi Y, Ikeuchi T, Murayama S, Ghetti B, Hasegawa M, Goedert M, Scheres SHW (2020). Novel tau filament fold in corticobasal degeneration. Nature.

[68] Zyśk M, Beretta C, Naia L, Dakhel A, Påvénius L, Brismar H, Lindskog M, Ankarcrona M, Erlandsson A (2023). Amyloid-β accumulation in human astrocytes induces mitochondrial disruption and changed energy metabolism. J Neuroinflammation.

