# Supplementary material for: Astrocytic accumulation of tau fibrils isolated from Alzheimer’s disease brains induces inflammation, cell-to-cell propagation and neuronal impairment
Source: Acta Neuropathol Commun. 2024 Feb 26;12:34. doi: 10.1186/s40478-024-01745-8 (PMC10898102; doi:10.1186/s40478-024-01745-8)
Supplement: Supplementary file 1 — Online Resource 1. List of all antibodies and dyes used in this study. [file 40478_2024_1745_MOESM1_ESM.pdf]

Table. S1 List of all antibodies and dyes used in this study

Manufacturer	Antibody/Dye	Target	Dilution
Sigma (P5282)	Phalloidin	Actin	ICC 1:1000
Abcam (ab20346)	VI-10	Vimentin	ICC 1:400
Abcam (ab4648)	Anti-GFAP	GFAP	ICC 1:400
Novus Biologicals (NBP1-87102)	S100B antibody	S100B	ICC 1:400
Novus Biologicals (NB100-1869)	EAAT1/GLAST-1/SLC1A3 Antibody	GLAST/EAAT1	ICC 1:400
Abcam (ab32127)	Anti-Synaptophysin	Synaptophysin	ICC 1:400
Biolegend (801202)	TUBB3	Tubulin β 3	ICC 1:400
Ebba biotech	Amytracker 680	Amyloid core of tau fibrils	ICC 1:1000
Thermo Fisher Scientific (A-11001, A-21422)	AlexaFluor Goat anti-Ms 488, 555	Mouse IgG	ICC 1:200
Thermo Fisher Scientific (A-11008, A-21245)	AlexaFluor Goat anti-Rb 488, 647	Rabbit IgG	ICC 1:200
	AlexaFluor Goat anti-Ch 647	Chicken IgY	ICC 1:200
Thermo Fisher Scientific (AHB0042)	Tau-5	Total tau	WB 1:400
Thermo Fisher Scientific (MN1020)	AT8	Phosphorylated tau Ser202, Thr205	WB 1:400

Thermo Fisher Scientific (35518)	Dylight 700	Mouse IgG	WB 1:20 000
Thermo Fisher Scientific (Sa5-535521)	Dylight 800	Mouse IgG	WB 1:20 000
