# Supplementary material for: Astrocytic accumulation of tau fibrils isolated from Alzheimer’s disease brains induces inflammation, cell-to-cell propagation and neuronal impairment
Source: Acta Neuropathol Commun. 2024 Feb 26;12:34. doi: 10.1186/s40478-024-01745-8 (PMC10898102; doi:10.1186/s40478-024-01745-8)
Supplement: Supplementary file 2 — Online Resource 2. Macro for quantification of astrocytes’ distant branching points. [file 40478_2024_1745_MOESM2_ESM.pdf]

/*

QUANTIFICATION OF TOTAL NUMBER OF BRANCHING POINTS

All threshold values (min and max) were set manually for each experiment and subsequently applied to all images.

/*

```
//Automation
inputfile = "C:\\Input\\";
outputfile = "C:\\Output\\";
list = getFileList(inputfile);
setBatchMode(true);
for (i=0; i<list.length; i++) {
showProgress(i+1, list.length);
open(inputfile+list[i]);

//Clear ROIs
run("Options...", "iterations=1 count=1 black do=Nothing");
roiManager("reset");


//Make 2x images to find edges
//Setting threshold and removing particles
run("Options...", "iterations=1 count=1 black edm=8-bit do=Nothing");
run("Duplicate...", " ");
run("8-bit");
rename("threshold");
run("Duplicate...", " ");
rename("edge");
run("Find Edges");
```

```
setAutoThreshold("Triangle dark");
run("Convert to Mask");
run("Grays");
setOption("BlackBackground", false);
setThreshold(255,255);
run("Analyze Particles...", "size=75-Infinity pixel show=Masks");
run("Grays");
rename("edge.particles");

//Blur image and remove particles
selectImage("threshold");
run("Gaussian Blur...", "sigma=0.8");
setAutoThreshold("Mean dark");
setThreshold(5, 255);
run("Convert to Mask");
run("Analyze Particles...", "size=75-Infinity pixel show=Masks");
run("Grays");
rename("threshold.particles");



//Skeletonize the cell and dilate/erode to size
run("Skeletonize (2D/3D)");
run("Create Selection");
roiManager("Add");
roiManager("select", 0);
run("Options...", "iterations=15 count=1 black edm=8-bit do=Dilate");
run("Options...", "iterations=15 count=2 black edm=8-bit do=Erode");
run("Skeletonize (2D/3D)");
rename("Skeleton");
run("Create Selection");
roiManager("Add");
```

```
//Count number of intersections
run("Convolve...", "text1=[1 1 1\n1 247 1\n1 1 1\n] normalize");
setThreshold(250,255);
run("Create Selection");
setSelectionName("Branchpoints");
roiManager("add");
run("Analyze Particles...", "size=1-Infinity pixel show=Masks display summarize add");
run("Close All");

                                        }
```

```
/*
QUANTIFICATION OF THE NUMBER OF BRANCHING POINTS IN THE SOMA


/*


//Automation
inputfile = "C:\\Input\\";
outputfile = "C:\\Output\\";;
list = getFileList(inputfile);
setBatchMode(true);
for (i=0; i<list.length; i++) {
 showProgress(i+1, list.length);
 open(inputfile+list[i]);

//Clear ROIs
run("Options...", "iterations=1 count=1 black do=Nothing");
roiManager("reset");


//Make 2x images to find edges
//Setting threshold and removing particles
run("Options...", "iterations=1 count=1 black edm=8-bit do=Nothing");
run("Duplicate...", " ");
run("8-bit");
rename("threshold");
run("Duplicate...", " ");
rename("edge");
run("Find Edges");
setAutoThreshold("Triangle dark");
```

```
run("Convert to Mask");
run("Grays");
setOption("BlackBackground", false);
setThreshold(255,255);
run("Analyze Particles...", "size=75-Infinity pixel show=Masks");
run("Grays");
rename("edge.particles");

//Blur image and remove particles
selectImage("threshold");
run("Gaussian Blur...", "sigma=0.8");
setAutoThreshold("Mean dark");
setThreshold(5, 255);
run("Convert to Mask");
run("Analyze Particles...", "size=75-Infinity pixel show=Masks");
run("Grays");
rename("threshold.particles");


//Skeletonize the cell and dilate/erode to size
run("Skeletonize (2D/3D)");
run("Create Selection");
roiManager("Add");
roiManager("select", 0);

run("Options...", "iterations=15 count=1 black edm=8-bit do=Dilate");
run("Options...", "iterations=15 count=2 black edm=8-bit do=Erode");

run("Skeletonize (2D/3D)");
rename("Skeleton");
run("Create Selection");
```

```
roiManager("Add");



//Removal of branchpoints in soma


selectImage("threshold");
run("Grays");
run("Distance Map");
setThreshold(15,255);
run("Create Selection");
roiManager("add");



selectImage("Skeleton");
roiManager("select", 2);
run("Clear Outside");

//Count number of intersections
run("Convolve...", "text1=[1 1 1\n1 247 1\n1 1 1\n] normalize");
setThreshold(250,255);
run("Create Selection");
setSelectionName("Branchpoints");
roiManager("add");
run("Analyze Particles...", "size=1-Infinity pixel show=Masks display summarize add");
run("Close All");


                    }
```

/*

To calculate the number of distant branching points, use the following formula:

NUMBER OF DISTANT BRANCHING POINTS=

TOTAL NUMBER OF BRANCHING POINTS - NUMBER OF BRANCHING POINTS IN THE SOMA

/*
