# Supplementary material for: Astrocytic accumulation of tau fibrils isolated from Alzheimer’s disease brains induces inflammation, cell-to-cell propagation and neuronal impairment
Source: Acta Neuropathol Commun. 2024 Feb 26;12:34. doi: 10.1186/s40478-024-01745-8 (PMC10898102; doi:10.1186/s40478-024-01745-8)
Supplement: Supplementary file 3 — Online Resource 3. Macro for quantification of intracellular Amytracker signal. [file 40478_2024_1745_MOESM3_ESM.pdf]

/*

QUANTIFICATION OF INTRACELLULAR AMYTRACKER SIGNAL

Definitions:

c1*= z-stack images.* ***c2****= Vimentin composite images.* ***c3****= Amytracker composite images.*

The threshold (*min* and *max*) is set manually for each experiment and subsequently applied to all images.

/*

```
//Automation
inputfile = "C:\\Input\\";
outputfile = "C:\\Output\\";
inputfilelist = getFileList(inputfile);
for(k=0;k<lengthOf(inputfilelist);k=k+2) {
            roiManager("Reset");
            open(inputfile + inputfilelist[k]);
            print(k);
            C2location=lastIndexOf(inputfilelist[k],"c2");
            print(C2location);
            name=inputfilelist[k];
            newname=replace(name, "c2", "c3");
            print(name);
            open(inputfile + newname);
            selectImage(name);


//Determination of cellular margins using vimentin signal
run("Set Scale...", "distance=1 known=0.65 pixel=1 unit=micrometer");
            run("Subtract Background...", "rolling=500");
            run("16-bit");
  setThreshold(min, max);
  run("Create Selection");
  roiManager("Add");
```

```
//Quantification of Amytracker signal
selectImage(newname);
    run("Set Scale...", "distance=1 known=0.65 pixel=1 unit=micrometer");
                    run("Subtract Background...", "rolling=50");
                    run("16-bit");
                    roiManager("Select", 0);
                    run("Clear Outside");
    roiManager("select", 0);
    setThreshold(min, max);
    run("Create Selection");
    run("Set Measurements...", "area mean integrated display redirect=None decimal=3");
run("Measure");
}
```
