# Supplementary material for: Astrocytic accumulation of tau fibrils isolated from Alzheimer’s disease brains induces inflammation, cell-to-cell propagation and neuronal impairment
Source: Acta Neuropathol Commun. 2024 Feb 26;12:34. doi: 10.1186/s40478-024-01745-8 (PMC10898102; doi:10.1186/s40478-024-01745-8)
Supplement: Supplementary file 4 — Online Resource 4. Macro for quantification of Synaptophysin-positive puncta. [file 40478_2024_1745_MOESM4_ESM.pdf]

/*

QUANTIFICATION OF SYNAPTOPHYSIN-POSITIVE PUNCTA PER CELL AREA

Definitions:

c1*= z-stack images.* ***c2****= Vimentin composite images.* ***c3****= Amytracker composite images.*

The threshold (*min* and *max*) and pixel size (*min* and *max*) are set manually for each experiment and subsequently applied to all images.

/*

// Counting the number of synaptophysin-positive puncta

```
//Automation

inputfile = "C:\\Input\\";

outputfile = "C:\\Output\\";;

list = getFileList(inputfile);

setBatchMode(true);

for (i=0; i<list.length; i++) {

 showProgress(i+1, list.length);

 open(inputfile+list[i]);


//Measurement

run("Set Scale...", "distance=1 known=0.65 pixel=1 unit=micrometer");

run("Subtract Background...", "rolling=50 sliding");

run("16-bit");

setAutoThreshold("Default dark");

setThreshold(min, max);

setOption("BlackBackground", true);

run("Convert to Mask");

run("Despeckle");

run("Watershed");

run("Analyze Particles...", "size=min-max pixel circularity=0.10-1.00 display summarize
add");


}
```

```
// Meaurement of cell area using β3-Tubulin signal

//Automation

inputfile = "C:\\Input\\";

outputfile = "C:\\Output\\";;

list = getFileList(inputfile);

setBatchMode(true);

for (i=0; i<list.length; i++) {

 showProgress(i+1, list.length);

 open(inputfile+list[i]);


//Measurement

run("Set Scale...", "distance=1 known=0.65 pixel=1 unit=micrometer");

run("Subtract Background...", "rolling=50 sliding");

run("16-bit");

setThreshold(min, max);

setOption("BlackBackground", true);

run("Set Measurements...", "area limit display redirect=None decimal=2");

run("Measure");


}
```
