# Supplementary material for: Astrocytic accumulation of tau fibrils isolated from Alzheimer’s disease brains induces inflammation, cell-to-cell propagation and neuronal impairment
Source: Acta Neuropathol Commun. 2024 Feb 26;12:34. doi: 10.1186/s40478-024-01745-8 (PMC10898102; doi:10.1186/s40478-024-01745-8)
Supplement: Supplementary file 5 — Online Resource 5. Transmission electron microscopy (TEM) measurements of tau fibrils. [file 40478_2024_1745_MOESM5_ESM.pdf]

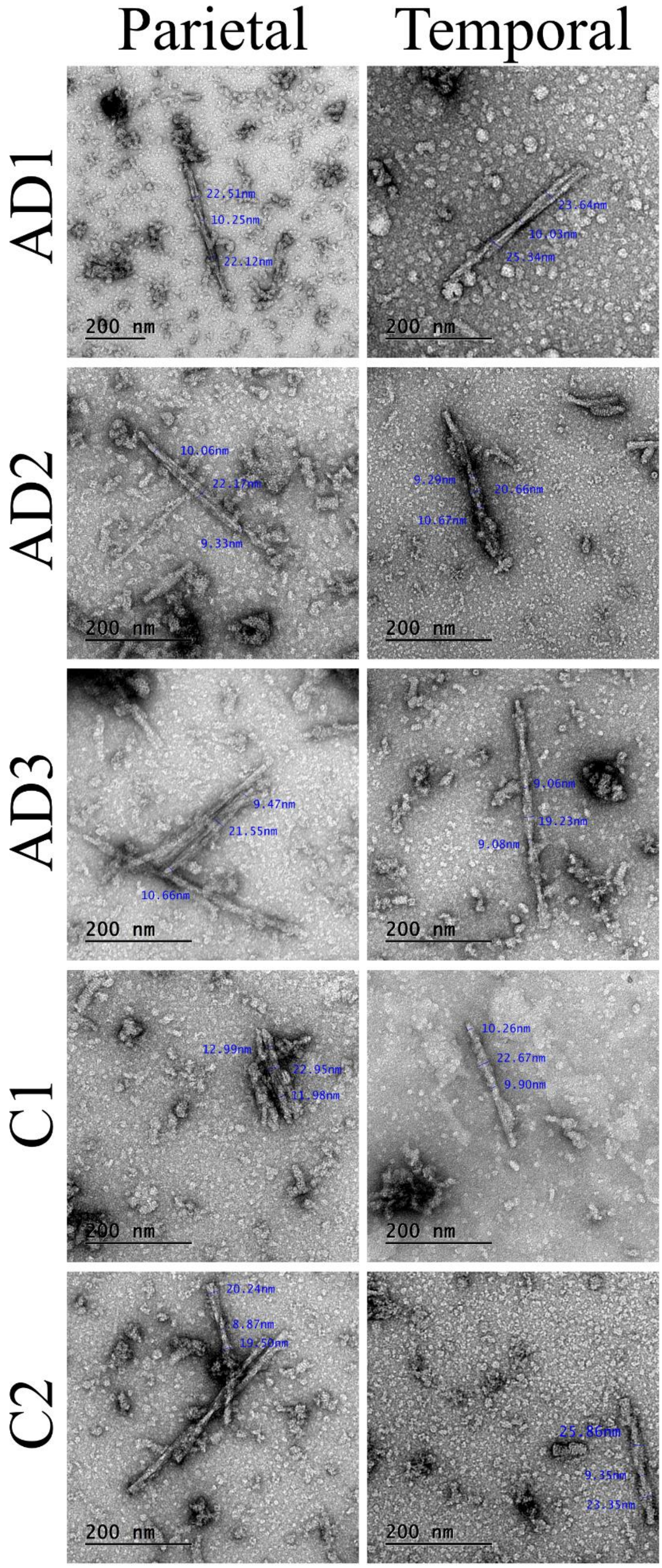

Online Resource 5 Transmission electron microscopy (TEM) measurements of tau fibrils confirmed the presence of paired helical filaments (PHFs) in all AD and control extracts. The width of fibrils is observed to alternate between approximately 10 and 20 nm, conforming with the previously reported description of PHFs (10).
