# Supplementary material for: Astrocytic accumulation of tau fibrils isolated from Alzheimer’s disease brains induces inflammation, cell-to-cell propagation and neuronal impairment
Source: Acta Neuropathol Commun. 2024 Feb 26;12:34. doi: 10.1186/s40478-024-01745-8 (PMC10898102; doi:10.1186/s40478-024-01745-8)
Supplement: Supplementary file 6 — Online Resource 6. Full western blots and total protein (No-Stain) blots. [file 40478_2024_1745_MOESM6_ESM.pdf]

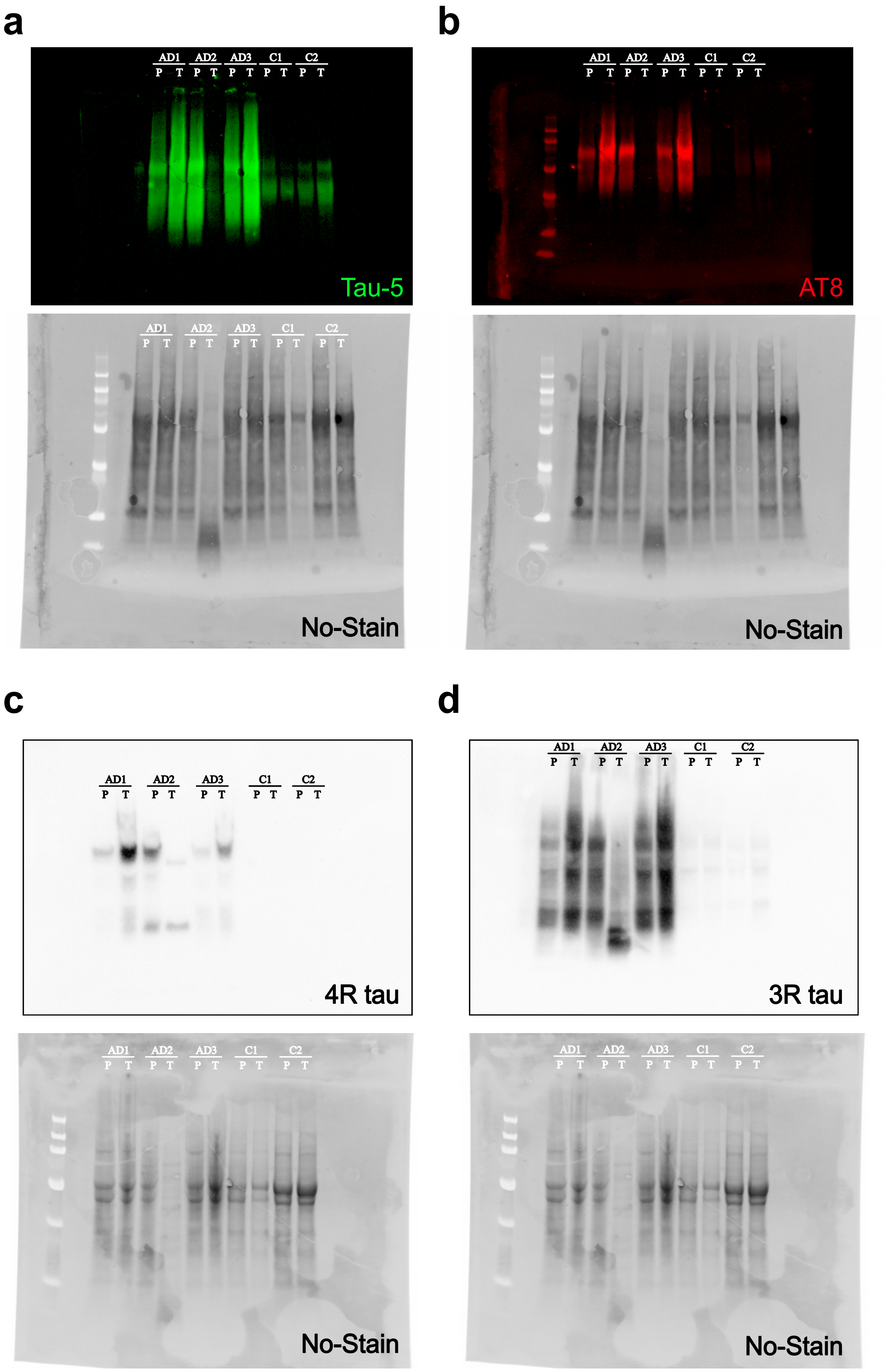

Online Resource 6 Full western blots and total protein (No-Stain) blots used to measure **(a)** total tau, **(b)** phosphorylated tau, **(c)** 4R tau and **(d)** 3R tau concentrations in brain tissue extracts. P= parietal. T=temporal.
