# Supplementary figures and images for: Astrocytic accumulation of tau fibrils isolated from Alzheimer’s disease brains induces inflammation, cell-to-cell propagation and neuronal impairment
Source: Acta Neuropathol Commun. 2024 Feb 26;12:34. doi: 10.1186/s40478-024-01745-8 (PMC10898102; doi:10.1186/s40478-024-01745-8)

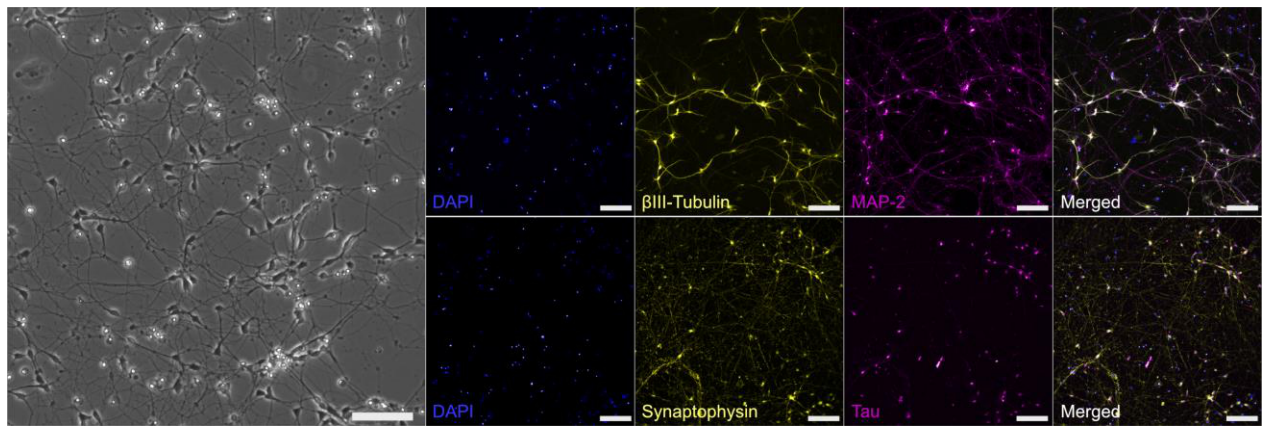

**Online Resource 13** Human iPSC derived neurons express cell type-specific markers. Scale bar= 100  $\mu$ m

Supplement: Supplementary file 7 — Online Resource 7. Human iPSC derived astrocytes express cell type-specific markers. [file 40478_2024_1745_MOESM7_ESM.pdf]

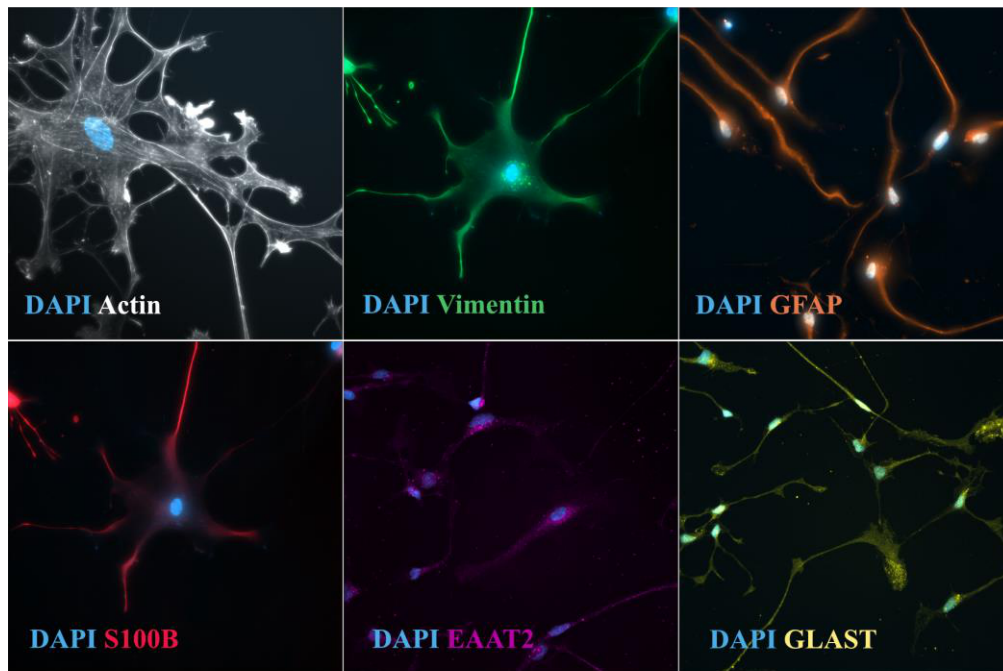

**Online Resource 7** Human iPSC derived astrocytes express cell type-specific markers.

Supplement: Supplementary file 8 — Online Resource 8. Fluorescent images and quantification of intracellular Amytracker signal in astrocytes. [file 40478_2024_1745_MOESM8_ESM.pdf]
