# Supplementary material for: Astrocytic accumulation of tau fibrils isolated from Alzheimer’s disease brains induces inflammation, cell-to-cell propagation and neuronal impairment
Source: Acta Neuropathol Commun. 2024 Feb 26;12:34. doi: 10.1186/s40478-024-01745-8 (PMC10898102; doi:10.1186/s40478-024-01745-8)
Supplement: Supplementary file 9 — Online Resource 9. Confocal imaging of phalloidin-stained human astrocytes demonstrating tunneling nanotube (TNT) formation. [file 40478_2024_1745_MOESM9_ESM.pdf]

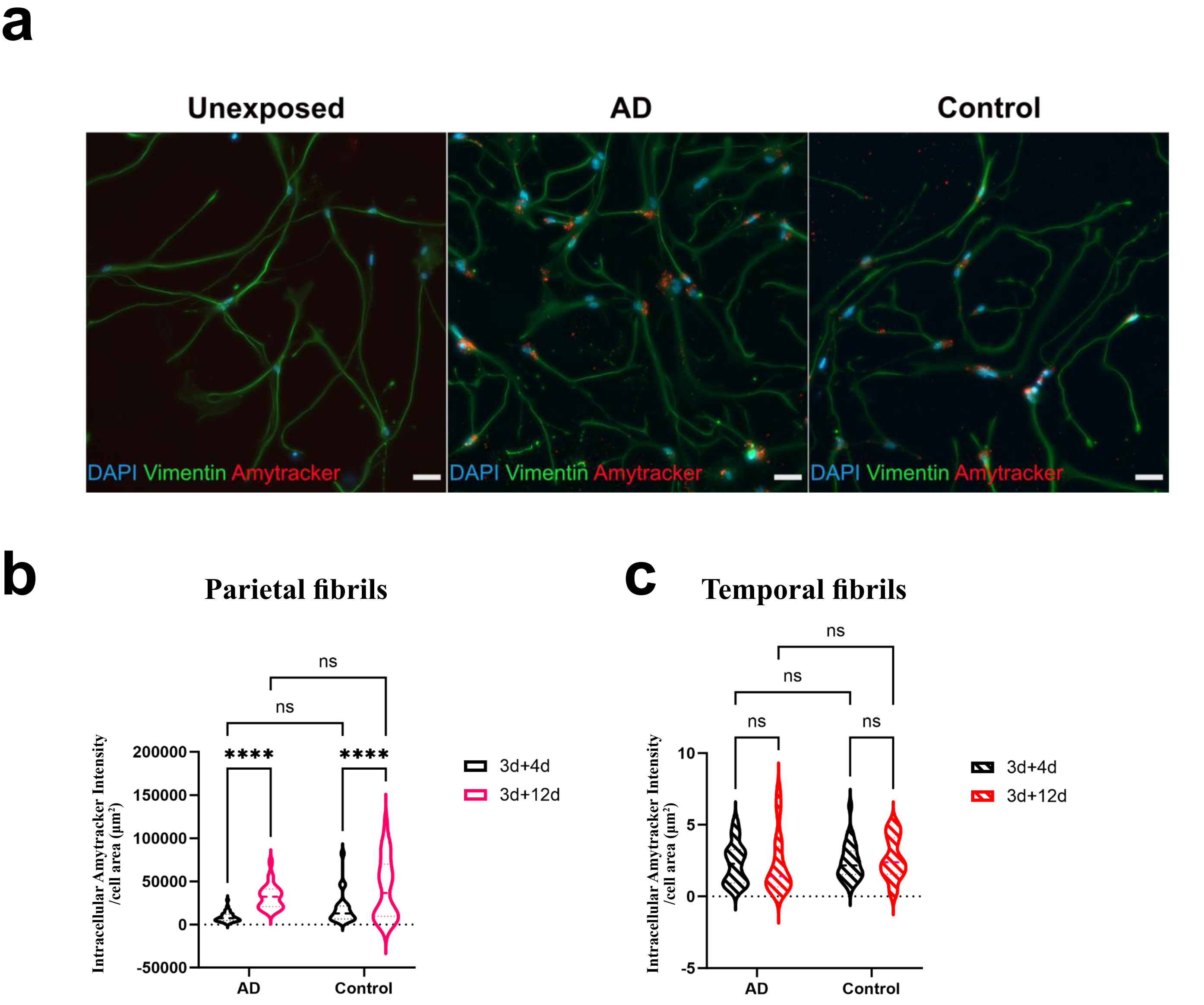

Online Resource 8 (**a**) Fluorescent images of Amytracker-stained astrocyte cultures demonstrating uptake of brain-derived AD and control tau fibrils by human astrocytes. No Amytracker signal was detected in unexposed astrocytes, excluding any endogenous formation of tau fibrils. Scale bar= 50 µm. (**b**) Quantification of intracellular Amytracker-680 signal in astrocytes exposed to parietal tau fibrils. (**c**) Quantification of intracellular Amytracker-680 signal in astrocytes exposed to temporal tau fibrils. Three AD and two control samples were used. N= 3 culture experiments, *n*= 10-12 non-overlapping fields per brain sample. Signal intensities in ***b*** and ***c*** were normalized to cell area (μm^2). The level of significance was set at $*p \leq 0.05$, $**p \leq 0.001$, $***p \leq 0.0001$, $****p \leq 0.00001$
