# Supplementary material for: Astrocytic accumulation of tau fibrils isolated from Alzheimer’s disease brains induces inflammation, cell-to-cell propagation and neuronal impairment
Source: Acta Neuropathol Commun. 2024 Feb 26;12:34. doi: 10.1186/s40478-024-01745-8 (PMC10898102; doi:10.1186/s40478-024-01745-8)
Supplement: Supplementary file 10 — Online Resource 10. Movie demonstrating tunneling nanotube (TNT)-mediated transfer of Amytracker-labelled tau fibril between human astrocytes. [file 40478_2024_1745_MOESM10_ESM.pdf]

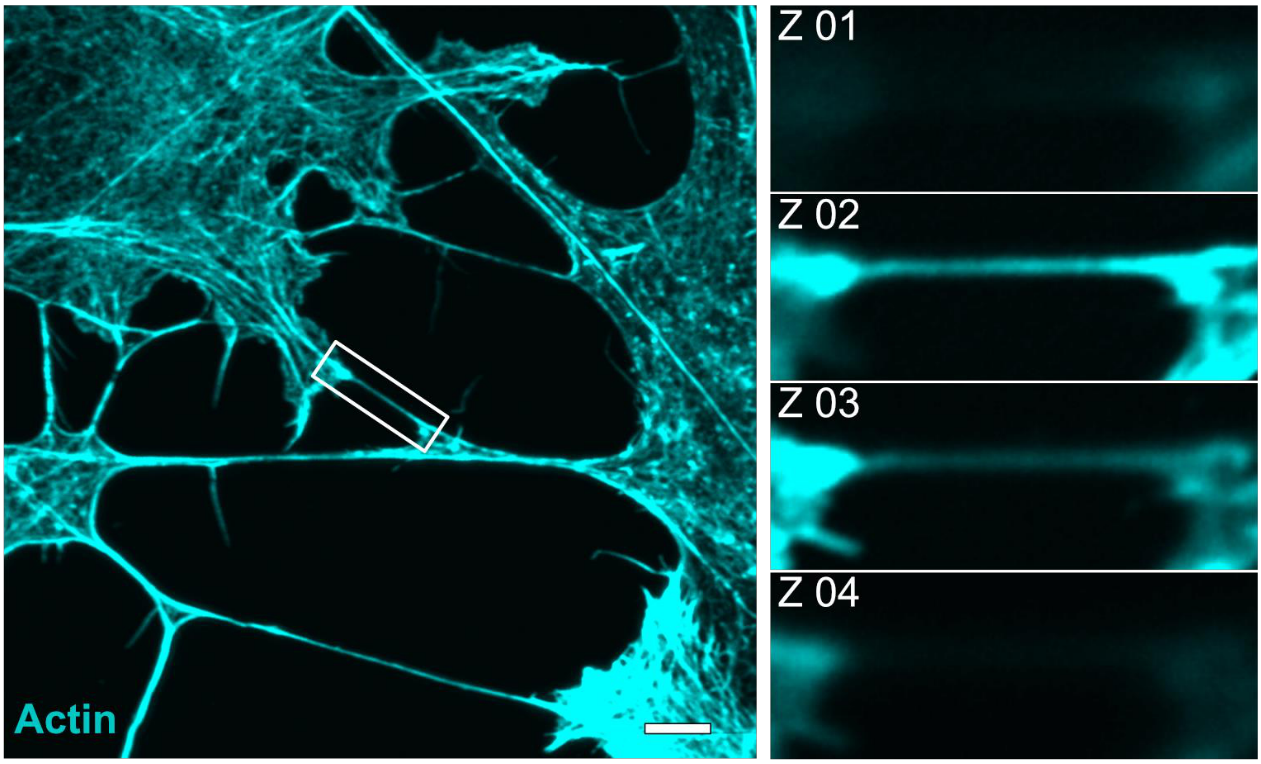

Online Resource 9 Confocal imaging of phalloidin-stained human astrocytes demonstrating tunneling nanotube (TNT) formation between two astrocytes. The different layers (Z 01-Z 04) of the Z-stack (of the white rectangle) are shown to the right. Scale bar= 5 µm
