# Supplementary material for: Astrocytic accumulation of tau fibrils isolated from Alzheimer’s disease brains induces inflammation, cell-to-cell propagation and neuronal impairment
Source: Acta Neuropathol Commun. 2024 Feb 26;12:34. doi: 10.1186/s40478-024-01745-8 (PMC10898102; doi:10.1186/s40478-024-01745-8)
Supplement: Supplementary file 12 — Online Resource 12. Confocal images of astrocyte-conditioned medium (ACM)-exposed biosensor HEK cells. [file 40478_2024_1745_MOESM12_ESM.pdf]

15:30

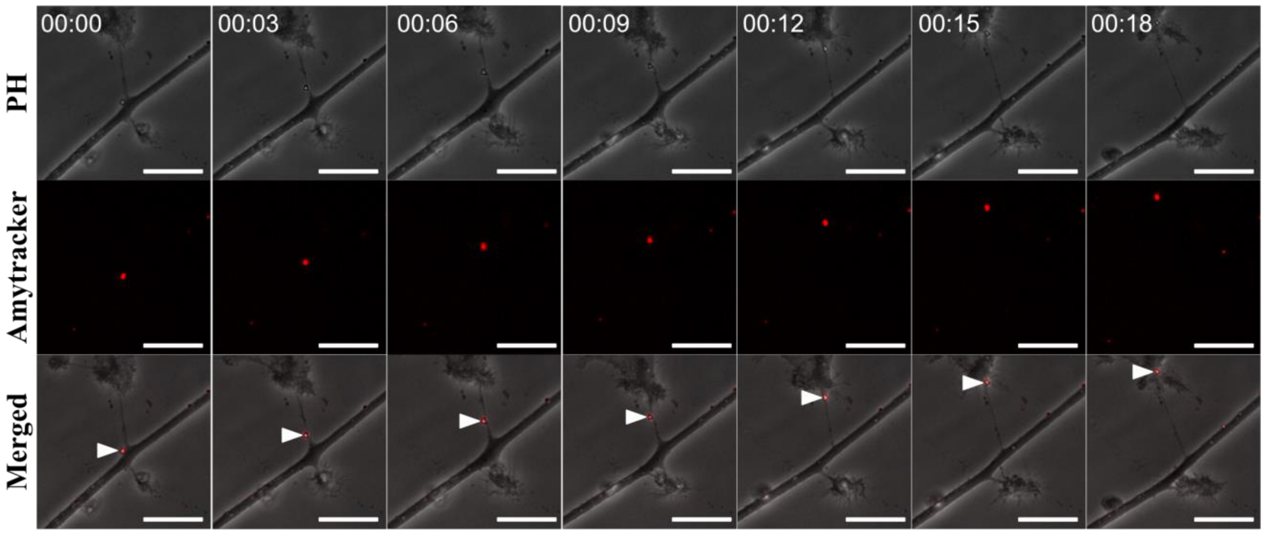

Online Resource 11 Brain derived AD tau fibrils are transferred between human astrocytes through tunneling nanotubes. Amytracker-labelled tau fibrils (arrowhead) travelling between two neighboring astrocytes. Images were captured with a 3-minutes interval. Scale bar= 25 μm
