# Supplementary material for: Astrocytic accumulation of tau fibrils isolated from Alzheimer’s disease brains induces inflammation, cell-to-cell propagation and neuronal impairment
Source: Acta Neuropathol Commun. 2024 Feb 26;12:34. doi: 10.1186/s40478-024-01745-8 (PMC10898102; doi:10.1186/s40478-024-01745-8)
Supplement: Supplementary file 13 — Online Resource 13. Human iPSC derived neurons express cell type-specific markers. [file 40478_2024_1745_MOESM13_ESM.pdf]

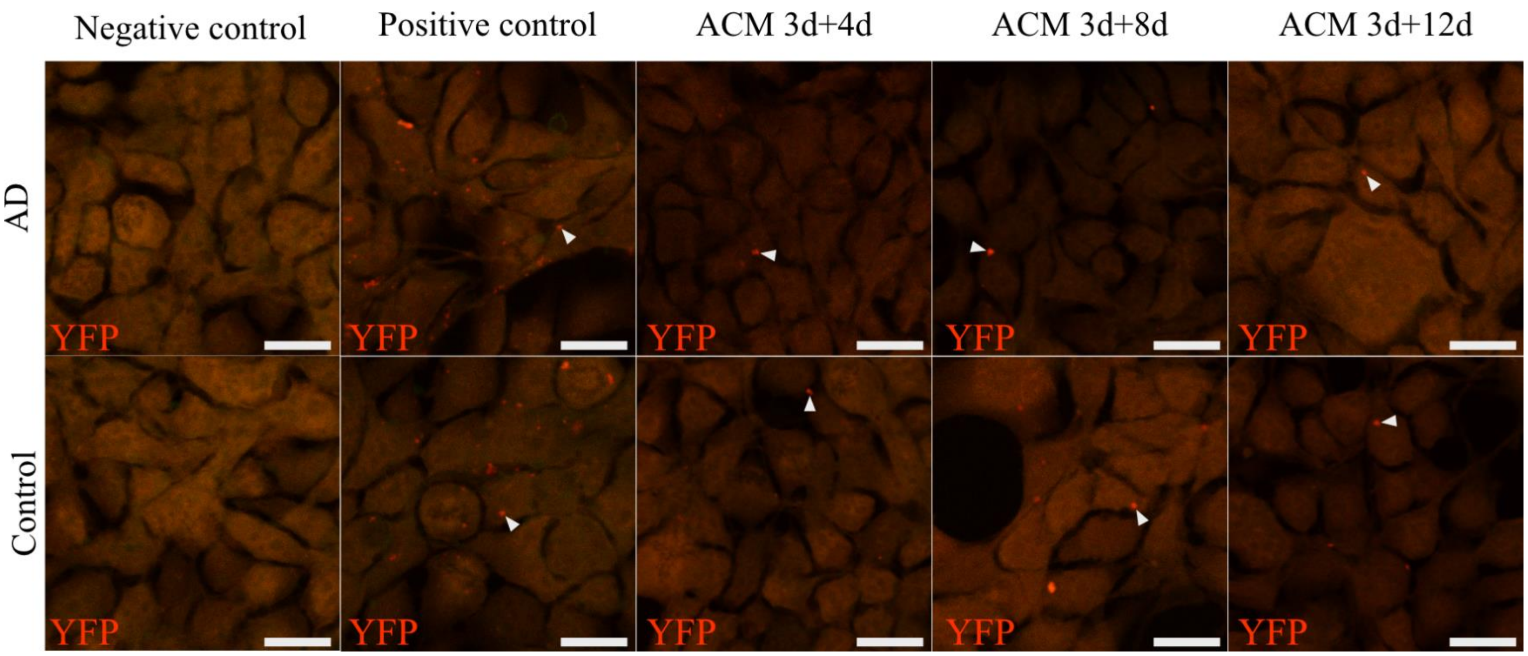

Online Resource 12 Representative confocal images of ACM-exposed biosensor HEK cells. Arrowheads indicate examples of positive FRET signal. Scale bar = 20 μm.
